# Cyclotrimerization Approach to Symmetric [9]Helical Indenofluorenes: Diverting Cyclization Pathways

**DOI:** 10.1002/chem.202301491

**Published:** 2023-07-21

**Authors:** Timothée Cadart, Tim Gläsel, Ivana Císařová, Róbert Gyepes, David Nečas, Marko Hapke, Martin Kotora

**Affiliations:** ^1^ Department of Organic Chemistry Faculty of Science Charles University in Prague Hlavova 8 128 43 Praha 2 Czech Republic; ^2^ Institute for Catalysis (INCA) Johannes Kepler University Linz Altenberger Strasse 69 A-4040 Linz Austria; ^3^ Department of Inorganic Chemistry Faculty of Science Charles University in Prague Hlavova 8 128 43 Praha 2 Czech Republic; ^4^ Department of Molecular Electrochemistry and Catalysis J. Heyrovský Institute of Physical Chemistry of the Czech Academy of Sciences Dolejškova 2155/3, 182 23 Praha 8 Czech Republic

**Keywords:** catalysis, C−C bond cleavage, cyclotrimerization, dispiroindenofluorenes, [9]helical structure

## Abstract

Catalytic cyclotrimerization routes to symmetrical [9]helical indenofluorene were explored by using different transition–metal complexes and thermal conditions. Depending on the reaction conditions, the cyclotrimerizations were accompanied by dehydro‐Diels–Alder reaction giving rise to another type of aromatic compounds. Structures of both symmetrical [9]helical cyclotrimerization product as well as the dehydro‐Diels–Alder product were confirmed by single‐crystal X‐ray diffraction analyses. Limits of enantioselective cyclotrimerization were assessed as well. DFT calculations shed light on the reaction course and the origin of diminished enantioselectivity.

## Graphical abstract

Catalytic cyclotrimerization (Rh, Co, and Ni catalysts) of triyne **1** gives rise preferentially to [9]helical indenofluorene, whereas it thermal cyclization gives rise to polyaromatic hydrocarbon **3**. The former reaction proceeds via classical alkyne cyclotrimerization, the latter via dehydro‐Diels–Alder reaction accompanied by unusual C−C cleavage. DFT calculations shed light on the course of the reaction.

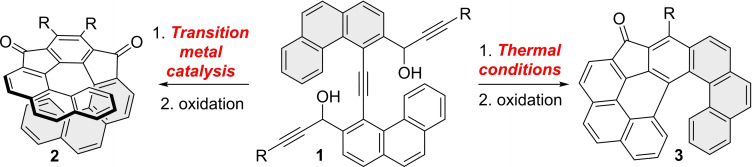

## Introduction

Helical compounds are an interesting class of substances possessing *ortho*‐fused rings and polyaromatic systems.^[1]^ Due to their twisted three‐dimensional shapes, caused by steric requirements bestowing unique inherent chirality on their molecules, they possess characteristic physical properties (optical and electronic). Such a property makes them interesting substrates for a potential application in material sciences and can be used also in asymmetric synthesis as ligands or organocatalysts.[^2^, ^3^]

As far as synthetic strategies to helical compounds are concerned, two approaches have been preferentially used: a) photocyclization reactions^[4]^ and b) transition metal‐catalyzed processes and among them catalytic [2+2+2] cyclotrimerization.^[5]^ Regarding the syntheses of larger helical compounds, it should be mentioned that photochemical cyclization were used for synthesis of [9]‐ and [16]helicenes[^6^, ^7^, ^8^, ^9^, ^10^, ^11^] and [*n*]heterohelicenes.^[12]^ Catalytic cyclotrimerization of alkynes was a basis for the syntheses of angular (helical) [6‐9]phenylenes,[^13^, ^14^] a [9]helicene,[^15^, ^16^, ^17^] a [11]helicene,^[18]^ [11]heterohelicenes,[^16^, ^19^, ^20^] a [19]oxahelicene,^[21]^ a hexapole [9]helicene,^[22]^ and a [17]heliphene.^[14]^

**Scheme 1:**
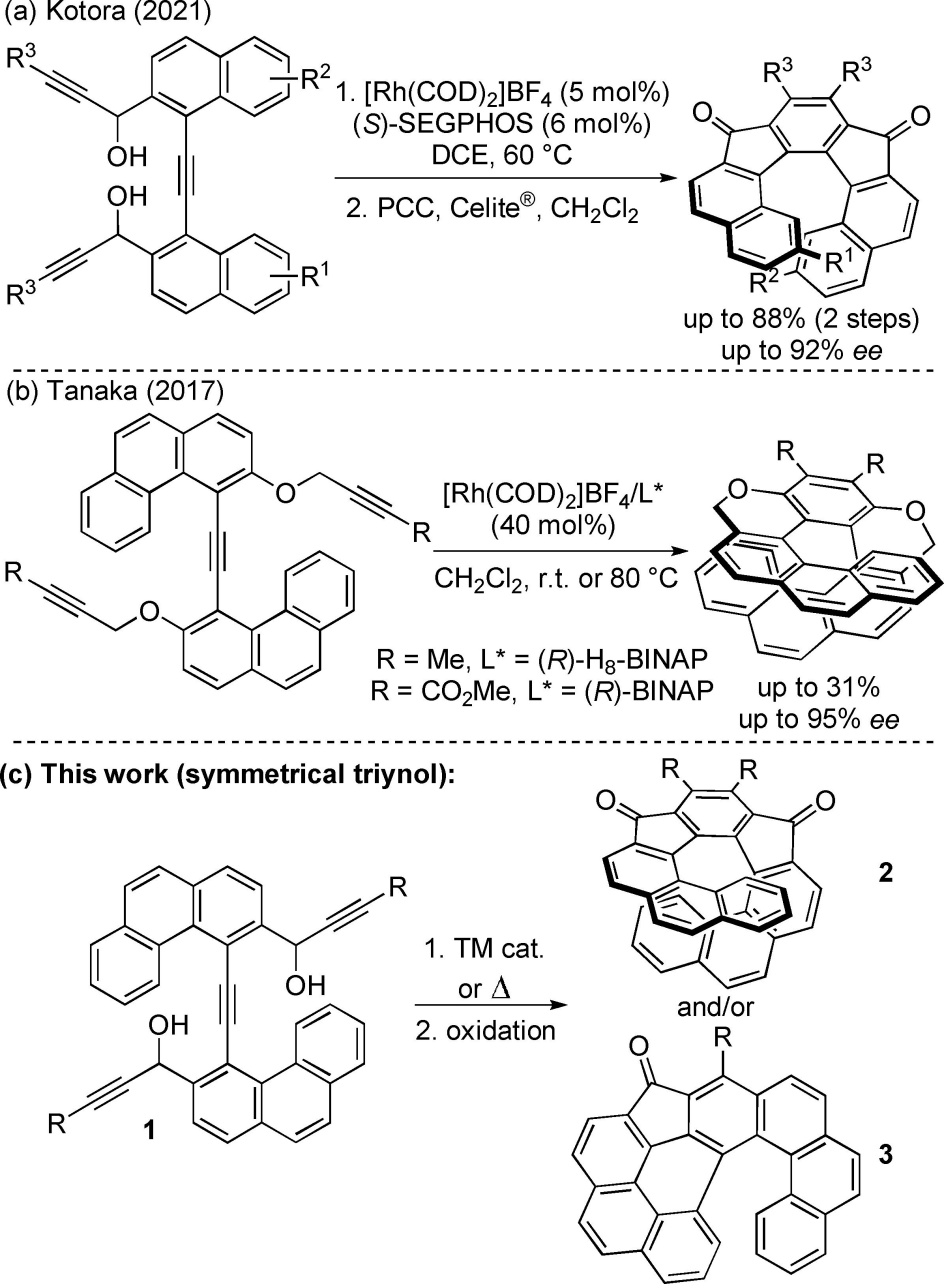
Cyclotrimerizations methods towards helical compounds.

During the last decade we have been interested in the application of catalytic [2+2+2] cycloaddition of alkynes for the synthesis of substances that possess selectively substituted fluorene scaffolds.[^23^, ^24^, ^25^] Our interest in such compounds stems not only from the synthetic point of view, but also because of their interesting photophysical properties. Recently, we have expanded this strategy also for preparation of [5]‐, [6]‐ and [7]helical substances with the indenofluorene (IF) scaffolds in very good yields.[^26^, ^27^] In the case of [5]‐ and [6]helical indenofluorenes, steric interaction of hydrogen atoms at the terminal aromatic rings conferred the products a helical shape, but due to a wider helical radius, these molecules are configurationally unstable in contrast to [5]helicene.[^28^, ^29^] In the case of [7]helical indenofluorenes increasing the size of the helical scaffold by two aromatic rings resulted in the overlap of the terminal carbon atoms of the edge of benzene rings, endowing them a reasonably high configurational stability. This led to the development of highly enantioselective synthesis of chiral [7]‐helical IFs (up to 92 % *ee*) with a reasonable configurational stability (Δ*G* ^#^=135.8 kJ mol^−1^) under mild reaction conditions employing a chiral Rh complex (Scheme 1a).^[30]^ Obviously, this success sparked our further interest to explore whether the cyclotrimerization approach could be extended also to the synthesis of larger (longer) configurationally stable [*n*]‐helical IFs and thus explore the limits of the cyclotrimerization methodology. In this respect, it has been already noted that formation of [9]helical molecules is usually accompanied by low yields and high catalyst loadings, as exemplified by the synthesis of [9]heterohelicene (Scheme 1b).^[17]^

Herein, we present experimental endeavors toward the synthesis of [9]helical indenofluorenes by using catalytic cyclotrimerization of the triynediol **1** bearing the phenanthrene moieties (Scheme 1c).

Although the project seemed to be initially a trouble‐free extension of the previous approach, it turned out that several factors (e. g., the presence metal ions, reaction temperature) determine the course of the reaction. The reaction proceeded either via the cyclotrimerization pathway forming [9]helical indenofluorenedione **2** and/or the dehydro‐Diels–Alder (DDA) pathway giving rise to **3**. Hence the essential part of the study was an effort to understand the crucial catalyst's role (Rh vs. Ni and Co catalytic systems), the structure of the substrate and reaction conditions leading to the desired helical indenofluorenedione **2** and/or to the DDA reaction product **3**. In addition, products were transformed into spirofluorene derivatives and their photophysical properties were determined.

## Results and Discussion

### Synthetic endeavors

**Scheme 2:**
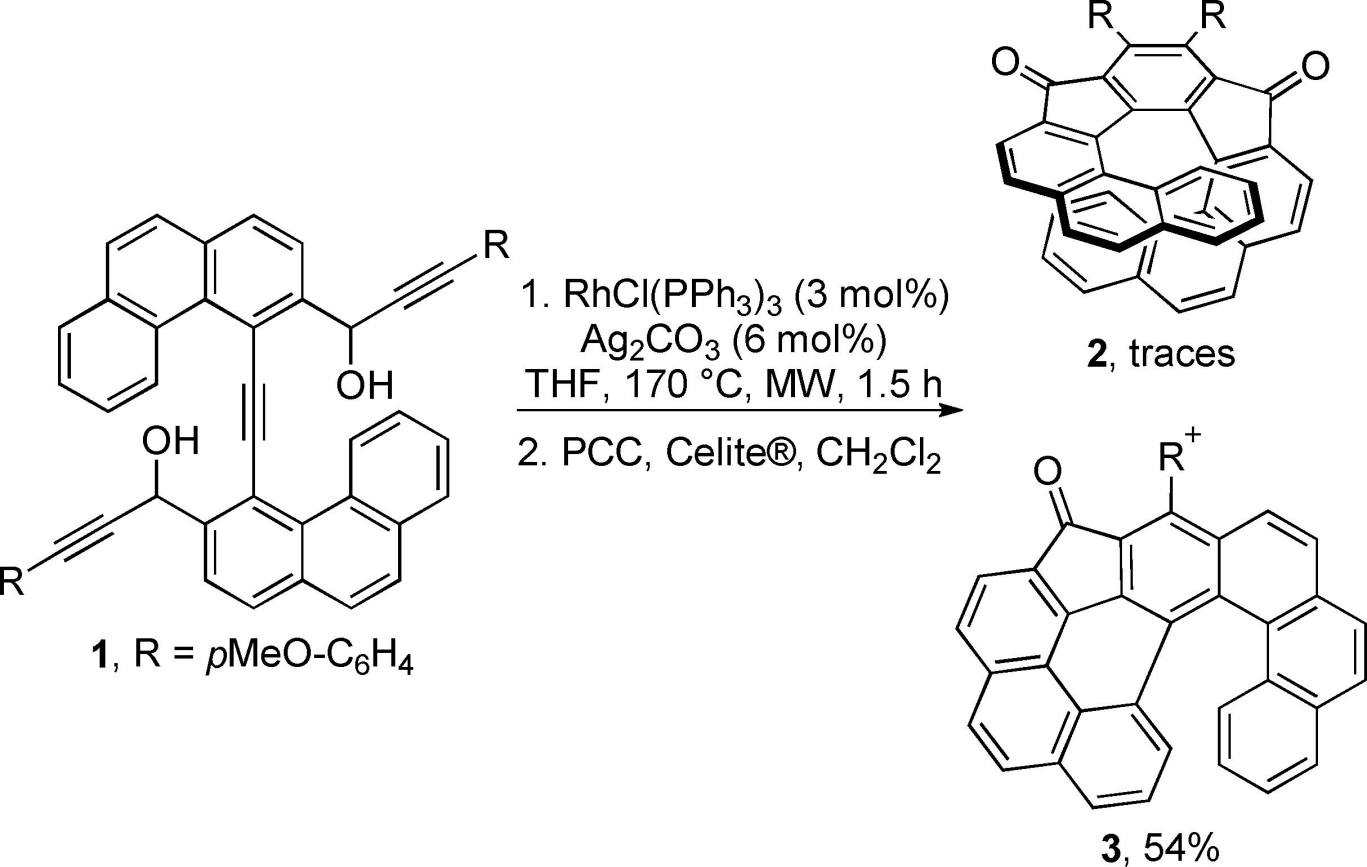
Cyclotrimerization of triynediol **1** catalyzed by the RhCl(PPh_3_)_3_/Ag_2_CO_3_ catalytic system.

At the outset, the triynediol **1** bearing phenanthrene moieties was prepared following the known procedures (see the Supporting Information). The triynediol **1** was subjected to cyclotrimerization conditions using catalytic amount of Wilkinson's catalyst (RhCl(PPh_3_)_3_) and silver carbonate in THF at 170 °C under microwave (MW) irradiation (Scheme 2).^[26]^ The obtained crude reaction mixture was directly oxidized with PCC in CH_2_Cl_2_ to avoid handling diastereoisomeric alcohols.^[30]^ Surprisingly, after isolation of the product (54 % yield) its NMR signals were in disagreement with those expected for 10,11‐bis(4‐methoxyphenyl)‐*as*‐indaceno[2,1‐*c*:7,8‐*c′*]diphenanthrene‐9,12‐dione **2** ([9]helical diketone). Further NMR and MS investigations of the isolated product, along with X‐ray analysis of a suitably grown single crystal revealed that the product was not the diketone indeed, but 6‐(4‐methoxyphenyl)‐5*H*‐benzo[*no*]indeno[2,1,7,6‐*ghij*]naphtho[1,2‐*a*]tetraphen‐5‐one **3** ([6]helical ketone **3**) bearing only one *p*‐methoxyphenyl moiety. Later, a more detailed ^1^H NMR analysis of the reaction mixture revealed that the cyclotrimerized product **2** was formed just in trace amounts (see Supporting Information for further details).

This unexpected reaction product prompted us to carry out detailed screening of reaction conditions (Table 1). First, we performed cyclotrimerizations with different catalysts to shed light on the course of the reaction. The catalysis by air‐stable and highly catalytically active CpCo[P(OEt)_3_](dmfu)^[31]^ and the “classical” catalyst CpCo(CO)_2_ furnished [9]helical diketone **2** and [6]helical ketone **3** after isolation in yields of 57 and 10 %, respectively (Table 1, entries 2 and 3). These results were rather surprising, because these catalysts often differ significantly in their catalytic performance with identical substrates.^[31b]^ In addition we have performed the reaction catalyzed by CpCo[P(OEt)_3_](dmfu) under irradiation conditions at high temperature, giving **2** and **3** in 36 and 51 % yields, respectively (Table 1 entry 4). Preferential formation of **3** may support a parallel radical pathway occurring under these conditions compared results from the thermal reaction conditions (see below). The use of Cp*RuCl(COD)^[32]^ and Ni(COD)(QD)^[33]^ resulted only in the formation of [9]helical diketone **2** in isolated yields of 50 and 70 %, respectively (Table 1, entries 5 and 6). ^1^H NMR analyses of the respective reaction mixture did not reveal the presence of **3** in either case. With these results in hand, our further attention focused back to the original reaction conditions. In this respect, cyclotrimerization with neat Wilkinson's catalyst (RhCl(PPh_3_)_3_) was attempted and gave rise to a mixture of **2** and **3** in a generally very high combined isolated yield of 88 % (Table 1, entry 7). The former product^[34]^ was isolated in 43 % yield whereas the latter one in 45 % yield. Then, we decided to exclude any transition metal catalyst and carried out the reaction under thermal conditions only and also in the presence of Ag_2_CO_3_ (Table 1, entries 8 and 9). The starting material was consumed in both cases but only [6]helical ketone **3** was detected in each reaction mixture and isolated in 45 and 56 % yields, respectively.

**Table 1:** Cyclotrimerization of **1** under different catalytic conditions.

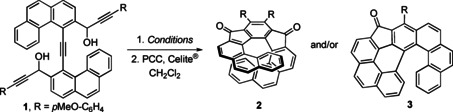					
Entry^[a]^	Catalytic system	Solvent	T [°C]	2 [%]^[b]^	3 [%]^[b]^
1^[c,d]^	RhCl(PPh_3_)_3_ (3 mol %), Ag_2_CO_3_ (6 mol %)	THF	170	traces	54
2	CpCo[P(OEt)_3_](dmfu) (5 mol %)	toluene	130	57	10
3	CpCo(CO)_2_ (5 mol %)	toluene	130	57	10
4	CpCo[P(OEt)_3_](dmfu) (5 mol %)	toluene	130	36	51
5	Cp*RuCl(COD) (5 mol %)	DCE	85	50	0
6	Ni(COD)(QD) (10 mol %), PPh_3_ (20 mol %)	toluene	100	70	0
7^[d]^	RhCl(PPh_3_)_3_ (3 mol %)	THF	170	43	45
8^[d]^	–	THF	170	0	45
9^[d]^	Ag_2_CO_3_ (6 mol %)	THF	170	0	56^[e]^

[a] Reactions were carried out on 0.1 mmol scale unless noted otherwise. [b] Isolated yield after oxidation reaction. [c] The reaction result provided for comparison. [d] The reaction was performed in a microwave reactor. [e] The reaction was performed on 1 mmol scale.

**Figure 1:**
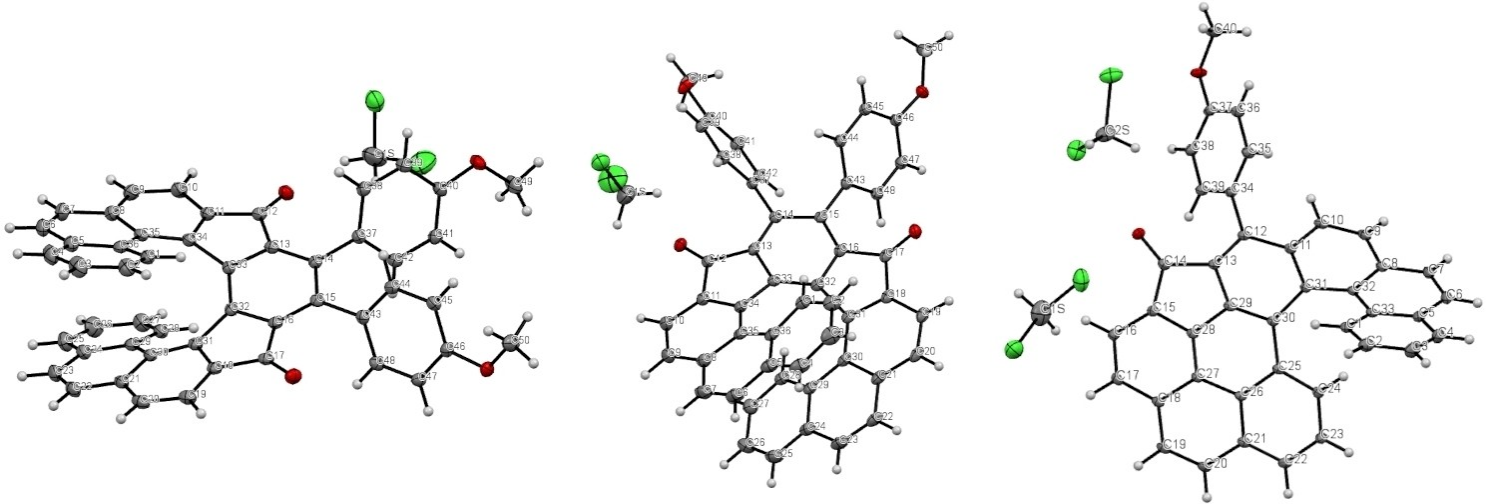
ORTEP drawing of [9]helical compound **2** (CCDC no. 2261993) side view (left) and top view (middle) and **3** (CCDC no. 2261994) (right).

Single crystals of **2** and **3** were obtained by pentane diffusion into their CH_2_Cl_2_ solutions and the respective crystal structures were determined by single‐crystal X‐ray diffraction analyses.^[35]^ The sums of seven dihedral angles, which should reflect the degree of molecular twist, is 120° for **2** (Figure 1). This value is considerably smaller than the one for the related [9]helicene (164.9°)^[11]^ indicating a lesser lead for the helix axial advance. Worth of mention is a very small value for ∠C034‐C033‐C032‐C031 that is mere 0.3° indicating only a very minute distortion of the central benzene ring.

Two units – the 3*H‐*benzo[*def*]cyclopenta[*jkl*]triphenylene and the [6]helicene scaffolds – could be easily recognized in the crystal structure of ketone **3** (Figure 1). The former scaffold is almost planar, whereas the latter has the typical helical arrangement. The sum of dihedral angles is only 74.5° which is by 12° lower that the value for [6]helicene (86.5°);^[36]^ however, it is closer to 79.3° observed for the recently prepared phenanthro[3,4,5,6‐*defgh*][6]helicene.^[37]^

### Mechanistic considerations

We assume that origin of the dichotomy in the catalytic and thermal reaction pathways could be caused by steric hindrance in the anticipated reaction intermediates. DFT computations on the tentative rhodacycloheptatriene intermediates (the anticipated last intermediates preceding the aromatic ring formation^[38]^) were carried out to clarify spatial arrangements and interactions of the helical fragment within these molecules. According to our results, the heptacyclic rhodacycloheptadiene intermediate becomes preferred thermodynamically over the nonacyclic rhodacycloheptadiene at 0 K (Table S2). In addition, a more suitable molecular shape for the formation of an initial adduct with [RhCl(PPh_3_)_2_] was suggested for the heptacycle (Table S3). Gibbs energies devised at 353.15 K suggested the preference for the heptacycle by −18.3 kJ mol^−1^ (specifically ΔG=−179.3 kJ mol^−1^ for the heptacycle and −161.0 kJ mol^−1^ for the nonacycle, Table S4). This energy difference indicates an increased steric repulsion for triyne **1**, which is less prone to react with the catalyst. The likelihood of the respective rhodacycloheptatriene formation becomes decreased and to overcome this obstacle, higher reaction temperatures are required. This finding provides a possible explanation also for other cases of catalytic cyclotrimerizations leading to larger helical substances, which call for employing higher reaction temperatures and/or high loads of catalysts for their successful completion.[^13^, ^14^, ^15^, ^16^, ^17^, ^18^, ^19^, ^20^, ^21^, ^22^] On the other hand, higher reaction temperatures can promote undesirable competing reactions, such as thermally induced DDA reaction of polyyne substrates leading to their transformations to other kinds of aromatic products.^[39]^

**Scheme 3:**
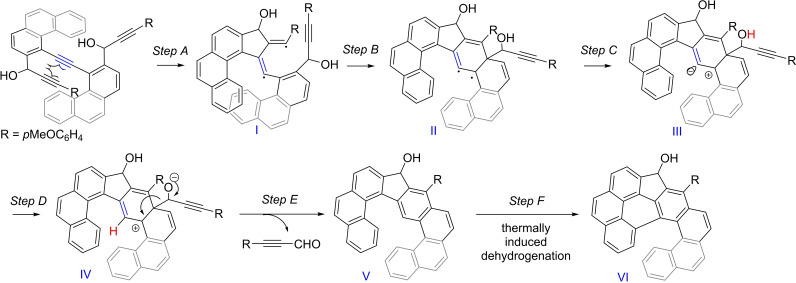
A tentative pathway for a DDA reaction pathway forming **3**.

As for the formation of **3**, we propose the following DDA reaction pathway^[39b]^ to rationalize the formation of ketone **3** through steps A−F (Scheme 3) that is based on our previous observations during preparation of unsymmetrical [7]indenofluorenes bearing the phenanthrene moiety.^[40]^ The first step involves thermally induced radical annulation of two alkyne moieties to form a dienyl diradical **I** (step A) in which one radical adds to the aromatic ring forming a new diradical **II** (step B). The ensuing electron transfer will give rise to the zwitterion **III**. Then, proton transfer from the propargyl hydroxyl group in **III** will take place giving rise to the zwitterion **IV** (step D) and its fragmentation will provide a propargyl aldehyde, which was detected in the reaction mixture (see Figure S3) and compound **V**. The intramolecular annulation in **V** to give leading to compound **VI** with the 1*H*‐indeno[2,1,7‐*cde*]pyren‐1‐ol scaffold is assumed to proceed thermally as the result of rather harsh reaction conditions (170 °C). Related intramolecular annulations of [n]helicenes have been reported recently, albeit under slightly different conditions.^[37]^ Although alcohol **VI** was isolated, it was difficult to remove accompanying impurities of similar polarity and to obtain it in an analytically pure form. Nonetheless, ^1^H and ^13^C NMR spectra provide sufficient evidence to support its proposed structure. Further evidence is provided by its HRMS analysis that showed a peak of 559.16696 m/z (C_40_H_24_O_2_Na^+^) (Figure S2).

### Synthesis of the spirofluorene and dispiro(indeno)fluorene scaffolds

**Scheme 4:**
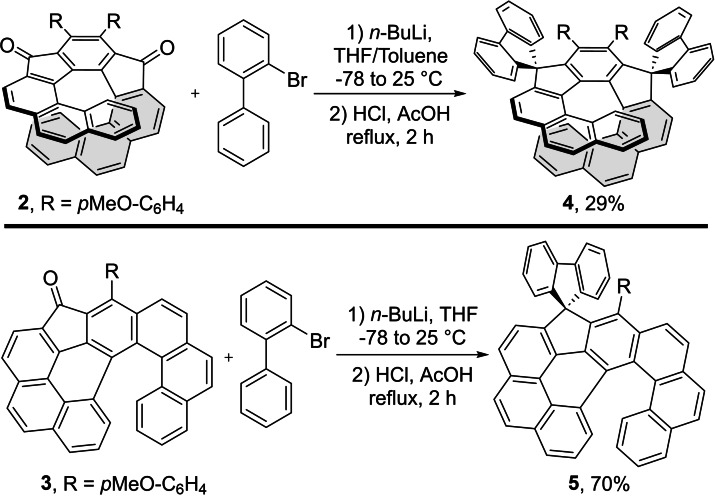
Conversion of ketones **2** and **3** to the respective bispiro and spiro derivatives **4** and **5**.

Diketone **2** and ketone **3** were converted to the corresponding spiro compounds **4** and **5** after the reaction with lithium biphenyl followed by acidic treatment (Scheme 4).[^41^, ^42^] In the former case toluene had to be used as the solvent of choice, because of a very low solubility of **2** in THF under given reaction conditions. Nevertheless, the final product **4** was isolated in mediocre yield of 29 %. On the other hand, conversion of **3** proceeded without any problems under the standard conditions, providing **5** with the efficient yield of 70 %.

### Photophysical properties

**Figure 2:**
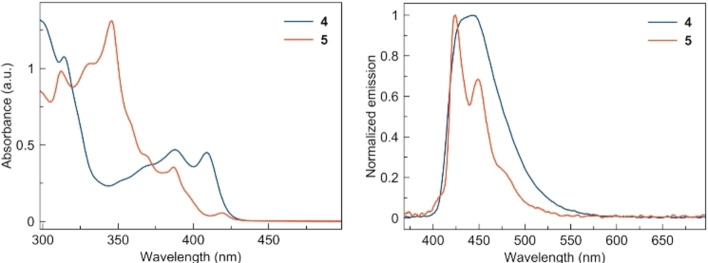
UV/Vis absorption (left) and normalized emission spectra (right) of **4** and **5** recorded in CH_2_Cl_2_.

Figure 2 shows the UV/Vis absorption and fluorescence spectra of **4** and **5** in dichloromethane. The UV/Vis absorption spectra differ perceptibly in molar attenuation coefficients with characteristic absorption bands at about 314, 352, 369, 388, and the lowest energy absorption around 409 nm for **4** and 312, 332, 346, 368, 387, and 419 for **5**. Both compounds behave as blue‐light fluorescent emitters. The emission maximum for **4** is λ_em_=443 nm (Φ_CL_=0.67). This value is red‐shifted by ∼10 nm in comparison with [7]helical bispiroindenofluorene (λ_em_=431 nm).^[30]^ The emission spectrum of **5** has two emission maxima at λ_em_=424 and 449 nm (Φ_CL_=0.17) and resemble the one observed for the recently prepared phenanthro[3,4,5,6‐*defgh*][6]helicene (λ_em_=∼488 and ∼512 nm).^[37]^

### Attempted enantioselective cyclotrimerization

The above‐mentioned results regarding steric interactions in the metallacycle intermediate, which were not present in the case of [7]‐helical indenofluorenes’ enantioselective synthesis, signaled potential hurdles for enantioselective cyclotrimerizations (Table 2). Nonetheless, we attempted to proceed with it by the previously used efficient Rh‐catalytic system composed of [Rh(COD)_2_](BF)_4_/(*S*)‐SEGPHOS (Entry 1). The conversion of the starting triyne **1** was very low at 80 °C (the required reaction temperature ensuring a high asymmetric induction in previous experiments) and the product was isolated in just 14 % yield with enantiopurity of mere 15 % *ee*, along with other unidentified aromatic products. As far as the configuration of the product is concerned, we assume it to be (*P*), like in the case of the [7]‐helical analog obtained with the same catalytic system. Surprisingly, using (*S*)‐H_8_‐BINAP at 60 °C under otherwise identical conditions gave no **2**, but **3** with 33 % yield (the result is not shown in Table 2). The Ir‐catalyzed reaction under the same conditions did not furnish the desired product (Entry 2). The use of a combination of Ni(COD)(QD) and (*R*)‐QUINAP (Entry 3) yielded **2** in high yield of 72 %, but with low enantioselectivity, which was again mere 12 % *ee* (the reaction carried out in THF did not proceed at all). Using catalytic systems based on Ni(COD)(QD) with (*R*,*R*)‐DIOP and MorfPhos also did not fare well. Albeit the former furnished **2** in 41 % yield, it did not instigated asymmetric induction (Table 2, entry 4). The latter catalytic system (Ni(COD)(QD)+MorfPhos) did not catalyze the reaction and the starting triyne **1** remained intact (Table 2, entry 5). Utilization of cobalt catalysis (CoBr_2_+phosphines) did not result in any product selectivity either. The use of a catalytic system comprising CoBr_2_ (25 mol %) and (*R*)‐QUINAP ligand (25 mol %) at 80 °C for 48 h only led to the formation of byproducts, the expected products **2** and **3** were not detected. However, increasing reaction temperature to 120 °C had a beneficial effect on the course of the reaction (Table 2, entries 6–8). Inspired by our earlier studies on asymmetric Co‐catalyzed cyclotrimerizations of triynes,^[43]^ the use of a catalytic system utilizing (*R*)‐QUINAP provided **2** in 33 % yield along with 18 % of **3** (Table 2, entry 6). Interestingly, when the *O*‐PINAP ligand (Table 2, entry 7) was used, exclusive formation of **3** was observed with an excellent yield of 89 %. It is remarkable that such a large shift in product selectivity compared to the structurally related ligand QUINAP was achieved. The selectivity for its formation was only matched by the use of silver carbonate with triyne **1** (Table 1, Entry 9). Using the (*S*,*R*_a_)‐Ph−Bn‐SIPHOS ligand provided the expected product **2** in mere 26 %, while **3** was formed in 50 % yield. Attempts to apply of (*S*)‐H_8_‐BINAP or (*R*,*R*)‐DIOP in the same concentration range with the CoBr_2_‐based catalytic system also led only to the formation of **3** and other byproducts.

**Table 2:** Attempted enantioselective cyclotrimerizations of **1** by transition metal catalysis.

Entry	Catalytic system^[a]^	Solvent	T [°C]^[b]^	**2** [%]^[c]^	*ee* [%]^[d]^	**3** [%]^[c]^
1	[Rh(COD)_2_](BF)_4_ (5 mol %), (*S*)‐SEGPHOS (6 mol %)	DCE	80	14	15	0
2	[Ir(COD)_2_](BF)_4_ (5 mol %), (*S*)‐SEGPHOS (6 mol %)	DCE	80	nd	nd	0
3	Ni(COD)(QD) (10 mol %), (*R*)‐QUINAP (20 mol %)	toluene	80	72^[e]^	12	0
4	Ni(COD)(QD) (10 mol %), (*R*,*R*)‐DIOP (20 mol %)	toluene	80	41^[f]^	0	0
5	Ni(COD)(QD) (10 mol %), (*S*)‐MorfPhos (20 mol %)	toluene	80	0	–	0
6	CoBr_2_ (25 mol %), (*R*)‐QUINAP (25 mol %)	THF	120	33^[g]^	0	18
7	CoBr_2_ (25 mol %), (*R*,*R*_a_)‐*O*‐PINAP (25 mol %)	THF	120	0	–	89
8	CoBr_2_ (25 mol %), (*S*,*R*_a_)‐Ph−Bn‐SIPHOS (25 mol %)	THF	120	26^[h]^	0	50

[a] Reactions were carried out on the 0.05 mmol scale for 20 h. [b] The cyclotrimerization didn't take place below 80 °C. [c] Isolated yield after oxidation. [d] The *ee* values were determined by HPLC using a column with a chiral stationary phase (see Supporting Information). [e] Full conversion of **1**. [f] 58 % conversion of **1**. [g] The formed product was not separable from side‐products. [h] Includes one not separable and identifiable side‐product.

The enantioselective cyclizations presumably suffered significantly from higher reaction temperatures, as they were in a number of cases also promoting the formation of byproducts (**3**), while failing to induct significant selectivity towards the desired product. Lower reaction temperatures, however, led to very slow or now reactions at all. It should be also mentioned that rather low asymmetric induction was observed during our previous attempts to synthesize enantioenriched unsymmetrical [7]helical indenofluorenes by cyclotrimerization of substrates bearing one phenanthrene moiety.^[40]^

## Conclusions

In summary, this study explored reaction pathways of triyne **1** conversions that can result either in the formation of [9]helical indenofluorene‐9,12‐dione **2**, under catalytic conditions, or to [6]helical ketone **3**, under thermal conditions. In addition, the obtained data provide clues why also in other catalytic cyclotrimerizations leading to larger helical compounds result in low yields and forcing reaction conditions. Structures of both products were unequivocally confirmed by single crystal X‐ray analyses. In addition, photophysical properties of the respective bispiro and spiro compounds were studied and determined. As far as the cyclotrimerization pathway is concerned, DFT calculations indicate that there is unfavorable steric interaction between the edge aromatic rings of the phenanthrene moiety and the hydroxy group(s) in the intermediate metallacycloheptatrienes. This might account for lower reactivity of the starting triyne **1** in comparison with the one used for synthesis of [7]helical indenofluorenes. Formation of a sterically demanding intermediate would require higher reaction temperatures to overcome it. The aforementioned factors have far‐reaching consequences for catalytic asymmetric induction which is rather low (if any) at temperatures required for successful cyclotrimerization. On the other hand, the higher reaction temperatures promote the competing DDA reaction diminishing the yields of the cyclotrimerization products. Nonetheless, the DDA reaction could open an alternative pathway to a new family of chiral helical nanographenes.^[37]^

In general, the unfavorable spatial arrangements (steric hindrance) in the intermediate species might be the reason why also other cyclotrimerization approaches to higher helicenes encounter problems and it is necessary to use high reaction temperatures (e. g. 250 °C was used for synthesis of [19]helicene^[21]^), high catalyst loadings, and special devices (e. g. a flow reactor) to carry them out.

## Experimental Section

**10,11‐Bis(4‐methoxyphenyl)‐as‐indaceno[2,1‐*c*:7,8‐*c*′]diphenanthrene‐9,12‐dione (2)**: Conditions in Table 1, Entry 6. A dry microwave vial was charged with Ni(COD)(QD) (0.01 mmol, 3.3 mg) and PPh_3_ (0.02 mmol, 5.2 mg) in toluene (2 mL). After 15 min of stirring, the corresponding triynediol **1** (0.1 mmol, 70 mg) was added to the solution. Afterwards, the reaction mixture was sealed and heated up to 100 °C for 20 h. Then it was cooled down to room temperatureand was concentrated under reduced pressure. The crude diols were directly oxidized to the corresponding diketones **2** without any further purification. To a solution of the crude diols in dry CH_2_Cl_2_ (5 mL) under argon atmosphere, pyridinium chlorochromate (0.3 mmol, 65 mg) and Celite^®^ (65 mg) were added. The resulting mixture was stirred at 25 °C for 3 h. Afterwards, the reaction mixture was filtered through a pad of 1 : 4 silica gel/Celite^®^. Then, the pad was washed using CH_2_Cl_2_ and the filtrate was concentrated under reduced pressure. Column chromatography of the residue on silica gel (8/1/1 to 6/1/1 hexanes/EtOAc/CH_2_Cl_2_) provided 48.6 mg (70 %) of the title compound as a reddish solid.

M.p.>370 °C. *R_f_* (6/1/1 hexanes/EtOAc/CH_2_Cl_2_)=0.32. ^1^H NMR (400 MHz; CD_2_Cl_2_) *δ*_H_ 7.79 (d, *J*=7.7 Hz, 2H), 7.59–7.52 (m, 4H), 7.36 (d, *J*=8.3 Hz, 2H), 7.32–7.22 (m, 4H), 7.12 (d, *J*=8.8 Hz, 2H), 7.07‐6.92 (m, 4H), 6.86–6.68 (m, 4H), 6.30 (ddd, *J*=8.3, 7.0, 1.4 Hz, 2H), 3.84 (s, 6H). ^13^C NMR (100 MHz; CD_2_Cl_2_) *δ*_C_ 190.6 (2 C), 159.5 (2 C), 144.4 (2 C), 142.2 (2 C), 141.8 (2 C), 137.3 (2 C), 136.94 (2 C), 136.89 (2 C), 133.6 (2 C), 131.6 (2 C), 131.2 (2 C), 130.1 (2 C), 129.4 (2 C), 128.2 (2 C), 128.1 (2 C), 127.6 (2 C), 127.0 (2 C), 126.7 (2 C), 124.9 (2 C), 123.2 (2 C), 120.1 (2 C), 113.2 (2 C), 113.1 (2 C), 55.5 (2 C). (One carbon signal is missing, probably cover by other signals.) IR (KBr) ν_max_ 3045, 3001, 2956, 2933, 2908, 2835, 1709, 1606, 1577, 1516, 1431, 1304, 1255, 1178, 1109, 1087, 1034, 856, 835, 816, 744 cm^−1^. HRMS (ESI+): m/z calcd for C_50_H_30_O_4_Na [(M+Na)^+^]: 717.20363, found: 717.20306.

**6‐(4‐Methoxyphenyl)‐5*H*‐benzo[*no*]indeno[2,1,7,6‐*ghij*]naphtho[1,2‐*a*]tetraphen‐5‐one (3)**: Conditions in Table 1, Entry 9. A dry microwave vial was charged with the triynediol **1** (1 mmol, 700 mg) and dissolved under argon atmosphere in dry THF (15 mL). After addition of Ag_2_CO_3_ (60 μmol, 17 mg), the reaction mixture was sealed and heated up to 170 °C for 1.5 h in a microwave reactor. Then it was cooled down to room temperature and the solvent was evaporated under reduced pressure. The crude diols were directly oxidized to the corresponding ketones without any further purification. To a solution of the crude diols in dry CH_2_Cl_2_ (50 mL) under argon atmosphere, pyridinium chlorochromate (3 mmol, 650 mg) and Celite^®^ (650 mg) were added. The resulting mixture was stirred at 25 °C for 3 h. Afterwards, the reaction mixture was filtered through a pad of 1 : 4 silica gel/Celite^®^. Then, the pad was washed using CH_2_Cl_2_ and the filtrate was concentrated under reduced pressure. Column chromatography of the residue on silica gel (8/1/1 to 6/1/1 hexanes/EtOAc/CH_2_Cl_2_) provided 299 mg (56 %) of the desired compound **3** as an orange solid.

M.p.=240‐242 °C (decomp). *R_f_* (6/1/1 hexanes/EtOAc/CH_2_Cl_2_)=0.4. ^1^H NMR (400 MHz; CDCl_3_) *δ*_H_ 8.20‐8.05 (m, 6H), 7.98–7.88 (m, 5H), 7.69 (d, *J*=8.0 Hz, 1H), 7.64‐7.50 (m, 2H), 7.35 (t, *J*=7.4 Hz, 1H), 7.28–7.24 (m, 1H), 7.17 (d, *J*=8.2 Hz, 2H), 6.74 (t, *J*=7.4 Hz, 1H), 3.98 (s, 3H). ^13^C NMR (100 MHz; CDCl_3_) *δ*_C_ 192.2, 160.0, 140.8, 138.9, 136.4, 135.2, 134.9, 133.4, 132.5, 131.93, 131.88, 131.8, 131.5, 131.0, 130.2, 130.1, 129.7, 129.4, 129.3, 128.3, 128.1, 128.0, 127.9, 127.6, 127.4, 126.8, 126.2, 126.1, 125.9, 125.8, 125.6, 125.4, 125.3, 124.7, 123.2, 121.9, 120.8, 113.9, 55.5. IR (KBr) ν_max_ 3041, 3014, 2991, 2962, 2925, 2850, 2833, 1709, 1657, 1618, 1608, 1583, 1570, 1525, 1508, 1464, 1373, 1288, 1246, 1219, 1176, 1107, 1092, 1038, 989, 837, 814, 739 cm^−1^. HRMS (ESI+): m/z calcd for C_40_H_22_O_2_Na [(M+Na)^+^]: 557.15120, found: 557.15114.

## Supporting Information

Experimental details, structural characterization, single crystal X‐ray structural analysis, UV‐Vis and emission spectroscopy, and theoretical calculation details, copies of ^1^H and ^13^C spectra. Additional references cited within the Supporting Information.[^44^, ^45^, ^46^, ^47^]

## Conflict of interest

The authors declare no conflict of interest.

1

## Supporting information

As a service to our authors and readers, this journal provides supporting information supplied by the authors. Such materials are peer reviewed and may be re‐organized for online delivery, but are not copy‐edited or typeset. Technical support issues arising from supporting information (other than missing files) should be addressed to the authors.

Supporting Information

## Data Availability

The data that support the findings of this study are available in the supplementary material of this article.
