# Supplementary material for: Cyclotrimerization Approach to Symmetric [9]Helical Indenofluorenes: Diverting Cyclization Pathways
Source: Chemistry. 2023 Jul 21;29(58):e202301491. doi: 10.1002/chem.202301491 (PMC10946996; doi:10.1002/chem.202301491)

Chemistry–A European Journal

Supporting Information

Cyclotrimerization Approach to Symmetric [9]Helical Indenofluorenes: Diverting Cyclization Pathways

Timothée Cadart, Tim Gläsel, Ivana Císařová, Róbert Gyepes, David Nečas, Marko Hapke,* and Martin Kotora*

1 General information **2**

2 Synthesis of starting material **3**

3 Cyclotrimerization with different transition metal catalysts. **6**

4 Enantioselective cyclotrimerizations **11**

5 Mechanistic investigations **14**

5.1 DFT Calculations 14

5.2 Reaction mixtures analyses 15

6 Synthesis of spirofluorene derivatives **18**

7 X-Ray diffraction analysis **20**

8 Copies of ^{1}H and ^{13}C NMR spectra **24**

1 General information

All reagents were commercially available and purchased from Sigma-Aldrich, Acros Organics, Fluorochem, Alfa Aesar, and Strem Chemicals companies. Solvents were purified and dried by distillation: tetrahydrofuran (THF) and toluene from sodium/benzophenone, dichloromethane and 1,2-dichloroethane from calcium hydride. Other solvents and all reagents were used without further purification. All reactions were performed under argon atmosphere unless otherwise noted. Chromatography column was performed on Silica gel 60A (40-60 μm) from Silicycle. Thin layer chromatography was performed on Silicycle silica gel 60 F_{254} pre-coated aluminum sheets. NMR spectra were recorded on Bruker AVANCE III Spectrometer (^{1}H at 400 MHz and ^{13}C at 101 MHz). All NMR spectra were measured in $CDCl_3$ or CD_2Cl_2 solutions and referenced to residual solvent signal: $CDCl_3$ (^{1}H, δ_H = 7.26; ^{13}C, δ_C = 77.16), CD_2Cl_2 (^{1}H, δ_H = 5.32; ^{13}C, δ_C = 53.84). Coupling constants *J* are given in Hz. The IR samples were recorded in KBr powder and measured on spectrometer Hermo Nicolet AVATAR 370 FT-IR and are reported in wave numbers (cm^{-1}). The MS spectra were recorded on an Agilent 5975 Inert MSD or GC×GC-TOFMS LECO Pegasus IVD device. All melting points are uncorrected and were determined on a Kofler apparatus KB T300. HPLC analyses were performed with YMC Chiral (4.6 mm × 250 mm) or Daicel Chiralpak® (4.6 mm × 250 mm) columns and carried out on an Ecom instrument using LCP4100 pump and LCD2083 UV detector. The values obtained from the enantiomeric ratio were rounded to the whole numbers.

2 Synthesis of starting material

4-Bromo-1,2-dihydrophenanthrene-3-carbaldehyde (S1). To a solution of dry DMF (37.2 mmol, 2.9 mL) in dry CH_2Cl_2 (50 mL), PBr_3 (31 mmol, 3 mL) was added dropwise at 0 °C. After 1 hour of stirring, the 2,3-dihydrophenanthren-4(1*H*)-one (12.4 mmol, 2.44 g) in CH_2Cl_2 (50 mL) was added. The mixture was stirring for 4 hours under reflux. Then, the reaction mixture was cooled to 0 °C and a saturated aqueous solution of $NaHCO_3$ (50 mL) was added. After the end of the gas generation, the layers were separated. The aqueous phase was extracted with CH_2Cl_2 (3 × 50 mL). The combined organic phases were washed with H_2O and dried over Na_2SO_4, filtered and concentrated under reduced pressure. Chromatography column of the residue on silica gel (11/1 hexanes/EtOAc) furnished 1.53 g (43%) of the title compound as a yellowish oil which solidified in time. ^{1}H NMR (400 MHz; $CDCl_3$) δ_H 10.32 (s, 1H), 8.71 (d, *J* = 8.7 Hz, 1H), 7.87-7.83 (m, 2H), 7.57 (ddd, *J* = 8.6, 6.8, 1.6 Hz, 1H), 7.50 (ddd, *J* = 8.1, 6.8, 1.2 Hz, 1H), 7.33 (d, J = 8.2 Hz, 1H), 2.85-2.82 (m, 2H), 2.61-2.57 (m, 2H). ^{13}C NMR (100 MHz; $CDCl_3$) δ_C 192.8, 141.0, 137.9, 135.7, 133.8, 131.9, 130.3, 130.2, 128.8, 126.4, 125.8, 125.4, 29.7, 22.7. The spectral data were in accordance with previously published results.[1]

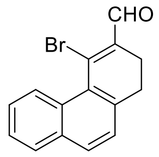

4-Bromophenanthrene-3-carbaldehyde (S2). A solution of **S1** (3.0 mmol, 880 mg) in dry Toluene (25 mL) was heated with DDQ (6.6 mmol, 1.5 g) at 120 °C and stirred for 16 hours. The mixture was diluted with hexanes, filtered and concentrated under reduced pressure. Chromatography column of the residue on silica gel (10/1 hexanes/EtOAc) furnished 633 mg (74%) of the title compound as a yellowish solid. ^{1}H NMR (400 MHz; $CDCl_3$) δ_H 10.79 (d, *J* = 0.8 Hz, 1H), 9.87-9.79 (m, 1H), 8.06 (d, *J* = 8.2 Hz, 1H), 7.97-7.89 (m, 1H), 7.89-7.84 (m, 2H), 7.75-7.65 (m, 3H). ^{13}C NMR (100 MHz; $CDCl_3$) δ_C 193.6, 138.6, 134.3, 133.9, 131.4, 130.3, 129.7, 129.0, 128.8, 127.81, 127.78, 126.6, 126.3, 126.2, 125.8. The spectral data were in accordance with previously published results.[1]

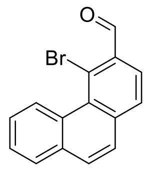

4-Ethynylphenanthrene-3-carbaldehyde (S3). In a Schlenck flask, $PdCl_2(PPh_3)_2$ (104 μmol, 73 mg), CuI (210 μmol, 40 mg) and aldehyde **S2** (2.08 mmol, 593 mg) were dissolved in THF (8.3 mL). To the solution, trimethylsilylacetylene (3.12 mmol, 435 μL) and Et_3N (8.3 mL) was added subsequently. The reaction mixture was stirring at reflux during 3 hours. Then, the mixture was cooled down to room temperature, filtered off on a pad of Celite®/silica and washed with Et_2O. The filtrate was concentrated under reduced pressure. The crude mixture was then, deprotected at 0 °C for 1 hour in MeOH with H_2O using K_2CO_3 pellets. The resulting mixture was neutralized with 1M HCl, extracted with Et_2O. Then, the combined layers were washed with H_2O, dried over Na_2SO_4, filtered and concentrated under reduced pressure. Chromatography column of the residue on silica gel (hexanes/EtOAc: 10/1) furnished 363 mg (76%) of the title compound as a yellowish solid.

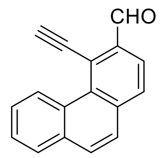

R_f (10/1 hexanes/EtOAc) = 0.32.

---

[1] M. W. Van der Meijden, E. Gelens, N. M. Quiros, J. D. Fuhr, J. E. Gayone, H. Ascolani, K. Wurst, M. Lingenfelder, R. Kellogg, *Chem. Eur. J.* **2016**, *22*, 1484-1492.

Mp = 116-121 °C.

^{1}H NMR (400 MHz; $CDCl_3$) δ_H 10.98 (d, J = 1.0 Hz, 1H), 10.37-10.34 (m, 1H), 8.14 (dd, J = 8.3, 1.4 Hz, 1H), 7.98-7.90 (m, 2H), 7.89 (d, J = 8.7 Hz, 1H), 7.74-7.68 (m, 3H), 4.15 (s, 1H).

^{13}C NMR (100 MHz; $CDCl_3$) δ_C 193.2, 137.1, 136.6, 133.4, 131.2, 130.9, 130.6, 130.3, 128.9, 127.8, 127.0, 126.7, 126.6, 123.9, 122.7, 92.3, 81.1.

IR (KBr) ν_{max} 3290, 3223, 3059, 2878, 2365, 1680, 1590, 1516, 1380, 1300, 1254, 1220, 1167, 1038, 850, 734, 521 cm^{-1}.

HRMS (EI+): *m/z* calcd for $C_{17}H_{10}O$: 230.0732, found: 230.0733.

4,4'-(Ethyne-1,2-diyl)bis(phenanthrene-3-carbaldehyde) (S4). In a flamed flask, $PdCl_2(PPh_3)_2$ (38.5 μmol, 27 mg), CuI (90 μmol, 15 mg), **S3** (0.77 mmol, 177 mg) and **S2** (0.77 mmol, 220 mg) were dissolved in THF (6.2 mL). To the solution, Et_3N (3.1 mL) was added. The reaction mixture was stirring at reflux during 3 hours. Then, the mixture was cooled down to room temperature, filtered off and washed with diethyl ether. The precipitate was then dissolved in CH_2Cl_2 and washed with H_2O (3 times), dried over Na_2SO_4, filtered and evaporated under reduce pressure to give 250 mg (75%) of the title compound with a homocoupling product in 1:0.1 ratio as a yellowish solid.

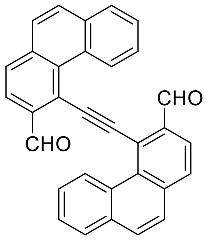

Mp = 159-164 °C.

^{1}H NMR (400 MHz; $CDCl_3$) δ_H 11.00 (s, 2H), 10.25 (d, J = 8.6 Hz, 2H), 8.24 (d, J = 8.2 Hz, 2H), 8.06 (d, J = 8.3, 0.9 Hz, 2H), 7.98-7.95 (m, 4H), 7.81 (d, J = 8.8 Hz, 2H), 7.64 (ddd, J = 8.0, 7.0, 1.1 Hz, 2H), 7.48 (ddd, J = 8.6, 7.0, 1.5 Hz, 2H).

^{13}C NMR (100 MHz; $CDCl_3$) δ_C 192.4 (2C), 136.9 (2C), 136.5 (2C), 133.6 (2C), 131.4 (2C), 130.8 (2C), 130.7 (2C), 129.2 (2C), 128.1 (2C), 127.2 (2C), 127.0 (2C), 126.3 (2C), 124.4 (2C), 124.2 (2C), 122.9 (2C), 100.5 (2C).

IR (KBr) ν_{max} 3049, 2857, 2354, 2326, 1687, 1649, 1586, 1307, 1254, 1227, 1097, 972, 846, 745 cm^{-1}.

HRMS (ESI+): m/z calcd for $C_{32}H_{18}O_2$ $[(M+Na)]^+$: 457.11990, found: 457.11993.

1,1'-(Ethyne-1,2-diylbis(phenanthrene-4,3-diyl))bis(3-(4-methoxyphenyl)prop-2-yn-1-ol) (1). *n*-BuLi (1.56 mmol, 1 mL) was added dropwise to a solution of 4-ethynylanisole (1.56 mmol, 190 μL) in anhydrous THF (12 mL) at -78 °C. After 30 min of stirring, the corresponding dialdehyde **S4** (0.52 mmol, 226 mg) was added. After the 5 min of stirring at -78 °C, the reaction mixture was warmed up to room temperature and stirred for 4 hours. Then, the resulting mixture was quenched using an aqueous solution of NH_4Cl, extracted with Et_2O (3 times). The organic layer was dried over Na_2SO_4, filtered and concentrated under reduced pressure. Chromatography column of the residue on silica gel (2/1 hexanes/EtOAc) provided 276 mg (76%) of the title compound (~1:1 mixture of diastereoisomers) as brownish solid.

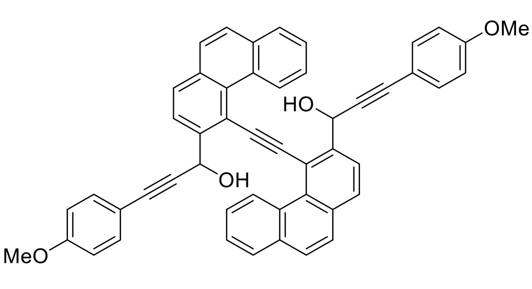

R_f (2/1 hexanes/EtOAc) = 0.18.

Mp = 125-130 °C (decomp).

^{1}H NMR (400 MHz; $CDCl_3$) δ_H 10.51 (d, J = 8.7 Hz, 1H), 10.41 (d, J = 8.7 Hz, 1H), 8.28 (d, J = 8.2 Hz, 1H), 8.19 (d, J = 8.2 Hz, 1H), 8.0-7.97 (m, 2H), 7.89-7.83 (m, 2H), 7.81-7.76 (m, 4H), 7.52-7.48 (m, 1H), 7.45-7.41 (m, 1H), 7.34-7.30 (m, 3H), 7.28-7.24 (m, 2H), 7.19-7.15 (m, 1H), 6.81 (s, 2H), 6.76-6.69 (m, 4H), 3.97 (br s, 1H), 3.74 (s, 3H), 3.73 (s, 3H), 3.25 (br s, 1H).
^{13}C NMR (100 MHz; $CDCl_3$) δ_C 159.81, 159.77, 143.9, 133.43, 133.36, 133.3, 133.2, 130.7, 130.62, 130.56, 130.53, 130.51, 130.4, 128.7, 128.6, 128.5, 127.3, 127.20, 127.19, 127.1, 126.8, 126.6, 126.4, 126.3, 125.6, 125.3, 117.8, 117.3, 114.6, 114.5, 113.94, 113.88, 100.04, 100.02, 87.9, 87.5, 87.4, 87.0, 64.3, 64.2, 55.33, 55.31.
IR (KBr) ν_{max} 3387, 3049, 2966, 2937, 2836, 2187, 1604, 1506, 1419, 1293, 1251, 1174, 1031, 954, 836, 748 cm^{-1}.
HRMS (ESI+): *m/z* calcd for $C_{50}H_{34}O_4Na$ [$(M+Na)^+$]: 721.23493, found: 721.23517.

3 Cyclotrimerization with different transition metal catalysts

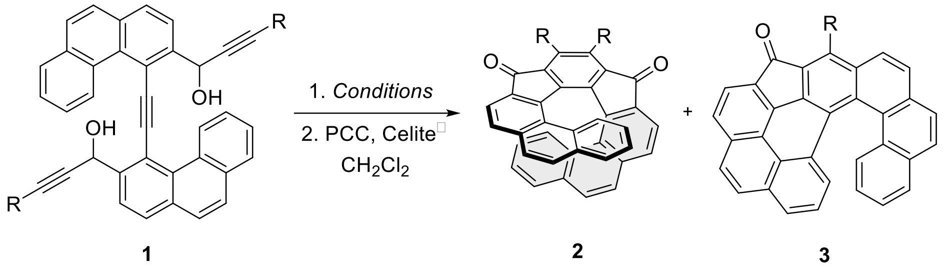

Rh-catalyzed cyclotrimerization ($RhCl(PPh_3)_3/Ag_2CO_3$, Table 1, Entry 1).
A dry microwave vial was charged with the triynediol **1** (0.25 mmol, 175 mg) and dissolved under argon atmosphere in THF (6 mL). After addition of Wilkinson's catalyst (7.5 µmol, 7 mg) and Ag_2CO_3 (15 µmol, 4.1 mg), the reaction mixture was sealed and heated up to 170 °C for 1.5 h in a microwave reactor. Afterwards, the reaction mixture was cooled down to room temperature and the solvent was evaporated under reduced pressure. Then, the crude diols were directly oxidized to the corresponding ketones without any further purification. To a solution of the crude diols in dry CH_2Cl_2 (15 mL) under argon atmosphere, pyridinium chlorochromate (0.75 mmol, 162 mg) and Celite® (160 mg) were added. The resulting mixture was stirred at 25 °C for 3 hours. Afterwards, the reaction mixture was filtered through a pad of 1:4 silica gel/Celite®. Then, the pad was washed using CH_2Cl_2 and the filtrate was concentrated under reduced pressure. Column chromatography of the residue on silica gel (8/1/1 to 6/1/1 hexanes/EtOAc/CH_2Cl_2) provided 72.7 mg (54%) of compound **3**.

Co-catalyzed cyclotrimerization (Table 1, Entries 2-3).
A dry microwave vial was charged with the corresponding triynediol **1** (0.1 mmol, 70 mg) and the corresponding cobalt catalyst (5 µmol) and dissolved in dry Toluene (2 mL). Afterwards, the reaction mixture was sealed and heated up to 130 °C for 20 h. Then, the reaction mixture was cooled down to room temperature and was concentrated under reduced pressure. The crude diols were directly oxidized to the corresponding ketones without any further purification. To a solution of the crude diols in dry CH_2Cl_2 (5 mL) under argon atmosphere, pyridinium chlorochromate (0.3 mmol, 65 mg) and Celite® (65 mg) were added. The resulting mixture was stirred at 25 °C for 3 hours. Afterwards, the reaction mixture was filtered through a pad of 1:4 silica gel/Celite®. Then, the pad was washed using CH_2Cl_2 and the filtrate was concentrated under reduced pressure. Column chromatography of the residue on silica gel (8/1/1 to 6/1/1 hexanes/EtOAc/CH_2Cl_2) provided 39.6 mg (57%) of the compound **2** and 5.4 mg (10%) of compound **3**.

Co-catalyzed cyclotrimerization under irradiation (Table 1, Entries 4).
A thermostated Schlenk-type reaction vessel was charged with triynediol **1** (0.025 mmol, 17.5 mg) and CpCo[P(OEt)$_3$](dmfu) (6.25 µmol, 2.7 mg) before dry toluene (3 mL) was added. The reaction mixture was heated to 120 °C for 16 h and was simultaneously irridiated using medium-pressure metal halide lamps (2*450 W). After cooling down, the crude diols were directly oxidized to the corresponding ketones without any further purification. To a solution

of the crude diols in dry CH_2Cl_2 (5 mL) under argon atmosphere, pyridinium chlorochromate (0.075 mmol, 16.3 mg) and Celite® (16.3 mg) were added. The resulting mixture was stirred at 25 °C for 3 hours. Afterwards, the reaction mixture was filtered through a pad of 1:4 silica gel/Celite®. Then, the pad was washed using CH_2Cl_2 and the filtrate was concentrated under reduced pressure. Column chromatography of the residue on silica gel (6/1/1 hexanes/EtOAc/CH_2Cl_2) provided 12.5 mg (36%) of compound **2** and 13.7 mg (51%) of compound **3**.

Ru-catalyzed cyclotrimerization (Table 1, Entry 5).

A dry microwave vial was charged with the corresponding triynediol **1** (0.1 mmol, 70 mg) and Cp*Ru(COD)Cl (5 µmol, 1.9 mg) and dissolved in dry DCE (2 mL). Afterwards, the reaction mixture was sealed and heated up to 85 °C for 20 h. Then, the reaction mixture was cooled down to room temperature and was concentrated under reduced pressure. The crude diols were directly oxidized to the corresponding diketones without any further purification. To a solution of the crude diols in dry CH_2Cl_2 (5 mL) under argon atmosphere, pyridinium chlorochromate (0.3 mmol, 65 mg) and Celite® (65 mg) were added. The resulting mixture was stirred at 25 °C for 3 hours. Afterwards, the reaction mixture was filtered through a pad of 1:4 silica gel/Celite®. Then, the pad was washed using CH_2Cl_2 and the filtrate was concentrated under reduced pressure. Column chromatography of the residue on silica gel (8/1/1 to 6/1/1 hexanes/EtOAc/CH_2Cl_2) provided 34.8 mg (50%) of the compound **2.**

Ni-catalyzed cyclotrimerization (Table 1, Entry 6).

A dry microwave vial was charged with Ni(COD)(QD) (0.01 mmol, 3.3 mg) and PPh_3 (0.02 mmol, 5.2 mg) in Toluene (2 mL). After 15 min of stirring, the corresponding triynediol **1** (0.1 mmol, 70 mg) was added to the solution. Afterwards, the reaction mixture was sealed and heated up to 100 °C for 20 h. The reaction mixture was cooled down to room temperature. Then, the reaction mixture was concentrated under reduced pressure. The crude diols were directly oxidized to the corresponding diketones **2** without any further purification. To a solution of the crude diols in dry CH_2Cl_2 (5 mL) under argon atmosphere, pyridinium chlorochromate (0.3 mmol, 65 mg) and Celite® (65 mg) were added. The resulting mixture was stirred at 25 °C for 3 hours. Afterwards, the reaction mixture was filtered through a pad of 1:4 silica gel/Celite®. Then, the pad was washed using CH_2Cl_2 and the filtrate was concentrated under reduced pressure. Column chromatography of the residue on silica gel (8/1/1 to 6/1/1 hexanes/EtOAc/CH_2Cl_2) provided 48.6 mg (70%) of the compound **2**.

Rh-catalyzed cyclotrimerization ($RhCl(PPh_3)_3$, Table 1, Entry 7).

A dry microwave vial was charged with the triynediol **1** (0.1 mmol, 70 mg) and dissolved under argon atmosphere in dry THF (6 mL). After addition of Wilkinson's catalyst (3 µmol, 2.8 mg), the reaction mixture was sealed and heated up to 170 °C for 1.5 h in a microwave reactor. The reaction mixture was cooled down to room temperature and the solvent was evaporated under reduced pressure. Then, the crude diols were directly oxidized to the corresponding ketones without any further purification. To a solution of the crude diols in dry CH_2Cl_2 (5 mL) under argon atmosphere, pyridinium chlorochromate (0.3 mmol, 65 mg) and Celite® (65 mg) were added. The resulting mixture was stirred at 25 °C for 3 hours. Afterwards, the reaction mixture

was filtered through a pad of 1:4 silica gel/Celite®. Then, the pad was washed using CH_2Cl_2 and the filtrate was concentrated under reduced pressure. Column chromatography of the residue on silica gel (8/1/1 to 6/1/1 hexanes/EtOAc/CH_2Cl_2) provided 29.9 mg (43%) of compound **2** and 24 mg (45%) of compound **3**.

Thermal reaction (Table 1, Entry 8).

A dry microwave vial was charged with the triynediol **1** (0.1 mmol, 70 mg) and dissolved under argon atmosphere in THF (6 mL). The reaction mixture was sealed and heated up to 170 °C for 1.5 h in a microwave reactor. Afterwards, the reaction mixture was cooled down to room temperature and the solvent was evaporated under reduced pressure. Then, the crude diols were directly oxidized to the corresponding ketones without any further purification. To a solution of the crude diols in dry CH_2Cl_2 (5 mL) under argon atmosphere, pyridinium chlorochromate (0.3 mmol, 65 mg) and Celite® (65 mg) were added. The resulting mixture was stirred at 25 °C for 3 hours. Afterwards, the reaction mixture was filtered through a pad of 1:4 silica gel/Celite®. Then, the pad was washed using CH_2Cl_2 and the filtrate was concentrated under reduced pressure. Column chromatography of the residue on silica gel (8/1/1 to 6/1/1 hexanes/EtOAc/CH_2Cl_2) provided 24 mg (45%) of compound **3**.

Thermal reaction in the presence of Ag_2CO_3 (Table 1, Entry 9).

A dry microwave vial was charged with the triynediol **1** (1 mmol, 700 mg) and dissolved under argon atmosphere in THF (15 mL). After addition of Ag_2CO_3 (60 µmol, 17 mg), the reaction mixture was sealed and heated up to 170 °C for 1.5 h in a microwave reactor. The reaction mixture was cooled down to room temperature and the solvent was evaporated under reduced pressure. Then, the crude diols were directly oxidized to the corresponding ketones without any further purification. To a solution of the crude diols in dry CH_2Cl_2 (50 mL) under argon atmosphere, pyridinium chlorochromate (3 mmol, 650 mg) and Celite® (650 mg) were added. The resulting mixture was stirred at 25 °C for 3 hours. Afterwards, the reaction mixture was filtered through a pad of 1:4 silica gel/Celite®. Then, the pad was washed using CH_2Cl_2 and the filtrate was concentrated under reduced pressure. Column chromatography of the residue on silica gel (8/1/1 to 6/1/1 hexanes/EtOAc/CH_2Cl_2) provided 299 mg (56%) of compound **3**.

10,11-Bis(4-methoxyphenyl)-as-indaceno[2,1-*c*:7,8-*c*']diphenanthrene-9,12-dione (2).

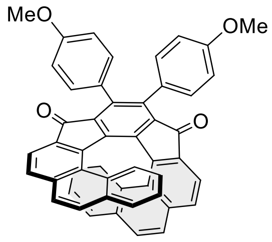

Following cyclotrimerization´s procedure (Table 1, Entry 6). A dry microwave vial was charged with Ni(COD)(QD) (0.01 mmol, 3.3 mg) and PPh_3 (0.02 mmol, 5.2 mg) in Toluene (2 mL). After 15 min of stirring, the corresponding triynediol **1** (0.1 mmol, 70 mg) was added to the solution. Afterwards, the reaction mixture was sealed and heated up to 100 °C for 20 h. The reaction mixture was cooled down to room temperature. Then, the reaction mixture was concentrated under reduced pressure. The crude diols were directly oxidized to the corresponding diketones **2** without any further purification. To a solution of the crude diols in dry CH_2Cl_2 (5 mL) under argon atmosphere, pyridinium chlorochromate (0.3 mmol, 65 mg) and Celite® (65 mg) were added. The resulting mixture was stirred at 25 °C for 3 hours. Afterwards, the reaction mixture was filtered through a pad of 1:4 silica gel/Celite®. Then, the pad was washed using CH_2Cl_2 and the filtrate was concentrated under reduced pressure. Column chromatography of the residue on silica gel (8/1/1 to 6/1/1 hexanes/EtOAc/CH_2Cl_2) provided 48.6 mg (70%) of the desired compound **2** as a reddish solid.

Mp > 370 °C.

R_f (6/1/1 hexanes/EtOAc/CH_2Cl_2) = 0.32.

^{1}H NMR (400 MHz; CD_2Cl_2) δ_H 7.79 (d, *J* = 7.7 Hz, 2H), 7.59-7.52 (m, 4H), 7.36 (d, *J* = 8.3 Hz, 2H), 7.32-7.22 (m, 4H), 7.12 (d, *J* = 8.8 Hz, 2H), 7.07-6.92 (m, 4H), 6.86-6.68 (m, 4H), 6.30 (ddd, *J* = 8.3, 7.0, 1.4 Hz, 2H), 3.84 (s, 6H).

^{13}C NMR (100 MHz; CD_2Cl_2) δ_C 190.6 (2C), 159.5 (2C), 144.4 (2C), 142.2 (2C), 141.8 (2C), 137.3 (2C), 136.94 (2C), 136.89 (2C), 133.6 (2C), 131.6 (2C), 131.2 (2C), 130.1 (2C), 129.4 (2C), 128.2 (2C), 128.1 (2C), 127.6 (2C), 127.0 (2C), 126.7 (2C), 124.9 (2C), 123.2 (2C), 120.1 (2C), 113.2 (2C), 113.1 (2C), 55.5 (2C). (One carbon signal is missing, probably cover by other signals.)

IR (KBr) ν_{max} 3045, 3001, 2956, 2933, 2908, 2835, 1709, 1606, 1577, 1516, 1431, 1304, 1255, 1178, 1109, 1087, 1034, 856, 835, 816, 744 cm^{-1}.

HRMS (ESI+): m/z calcd for $C_{50}H_{30}O_4Na$ [(M+Na)$^+$]: 717.20363, found: 717.20306.

6-(4-Methoxyphenyl)-5*H*-benzo[*no*]indeno[2,1,7,6-*ghij*]naphtho[1,2-*a*]tetraphen-5-one (3). Following reaction´s procedure (Table 1, Entry 9). A dry microwave vial was charged with the triynediol **1** (1 mmol, 700 mg) and dissolved under argon atmosphere in dry THF (15 mL). After addition of Ag_2CO_3 (60 μmol, 17 mg), the reaction mixture was sealed and heated up to 170 °C for 1.5 h in a microwave reactor. The reaction mixture was cooled down to room temperature and the solvent was evaporated under reduced pressure. Then, the crude diols were directly oxidized to the corresponding ketones without any further purification. To a solution of the crude diols in dry CH_2Cl_2 (50 mL) under argon atmosphere, pyridinium chlorochromate (3 mmol, 650 mg) and Celite® (650 mg) were added. The resulting mixture was stirred at 25 °C for 3 hours. Afterwards, the reaction mixture was filtered through a pad of 1:4 silica gel/Celite®. Then, the pad was washed using CH_2Cl_2 and the filtrate was concentrated under reduced

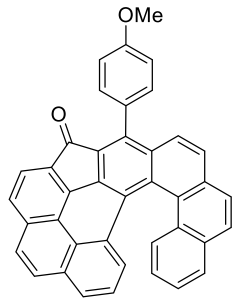

pressure. Column chromatography of the residue on silica gel (8/1/1 to 6/1/1 hexanes/EtOAc/CH_2Cl_2) provided 299 mg (56%) of the desired compound **3** as an orange solid.

Mp = 240-242 °C (decomp).

R_f (6/1/1 hexanes/EtOAc/CH_2Cl_2) = 0.4.

^{1}H NMR (400 MHz; $CDCl_3$) δ_H 8.20-8.05 (m, 6H), 7.98-7.88 (m, 5H), 7.69 (d, J = 8.0 Hz, 1H), 7.64-7.50 (m, 2H), 7.35 (t, J = 7.4 Hz, 1H), 7.28-7.24 (m, 1H), 7.17 (d, J = 8.2 Hz, 2H), 6.74 (t, J = 7.4 Hz, 1H), 3.98 (s, 3H).

^{13}C NMR (100 MHz; $CDCl_3$) δ_C 192.2, 160.0, 140.8, 138.9, 136.4, 135.2, 134.9, 133.4, 132.5, 131.93, 131.88, 131.8, 131.5, 131.0, 130.2, 130.1, 129.7, 129.4, 129.3, 128.3, 128.1, 128.0, 127.9, 127.6, 127.4, 126.8, 126.2, 126.1, 125.9, 125.8, 125.6, 125.4, 125.3, 124.7, 123.2, 121.9, 120.8, 113.9, 55.5.

IR (KBr) ν_{max} 3041, 3014, 2991, 2962, 2925, 2850, 2833, 1709, 1657, 1618, 1608, 1583, 1570, 1525, 1508, 1464, 1373, 1288, 1246, 1219, 1176, 1107, 1092, 1038, 989, 837, 814, 739 cm^{-1}.

HRMS (ESI+): m/z calcd for $C_{40}H_{22}O_2Na$ [$(M+Na)^+$]: 557.15120, found: 557.15114.

4 Enantioselective cyclotrimerizations

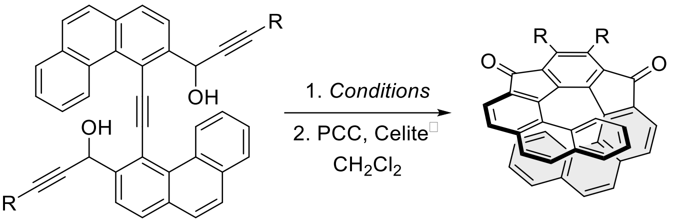

1, R = *p*MeO-C_6H_4 **2**

Rh/(*S*)-SEGPHOS complex-catalyzed reaction (Table 2, Entry 1).
A dry microwave vial was charged with $[Rh(COD)_2]BF_4$ (5 μmol, 2.0 mg) and (*S*)-SEGPHOS® (0.06 mmol, 3.7 mg) in dry DCE (2 mL) under argon atmosphere. H_2 gas was bubbled into the reaction mixture for 15 min.[2] Afterwards, the corresponding triynediol **1** (0.1 mmol, 70 mg) was added under argon atmosphere. The reaction was stirred at 80 °C for 24 hours. Then, the reaction mixture was concentrated under reduced pressure. The crude diols were directly oxidized to the corresponding diketone **2** without any further purification. To a solution of the crude diols in dry CH_2Cl_2 (5 mL) under argon atmosphere, pyridinium chlorochromate (0.3 mmol, 65 mg) and Celite® (65 mg) were added. The resulting mixture was stirred at 25 °C for 3 hours. Afterwards, the reaction mixture was filtered through a pad of 1:4 silica gel/Celite®. Then, the pad was washed using CH_2Cl_2 and the filtrate was concentrated under reduced pressure. Column chromatography of the residue on silica gel provided products **2**.
HPLC analysis: 57.5:42.5 e.r. (column YMC CHIRAL Cellulose-SB (Heptane/*i*-PrOH = 80/20, flow rate 0.7 mL/min, UV 254 nm, t_{major} = 21.8 min; t_{min} = 26.0 min).

Racemic			(*S*)-SEGPHOS®, **57.5:42.5 e.r**		
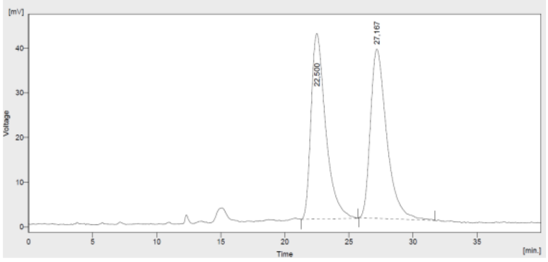			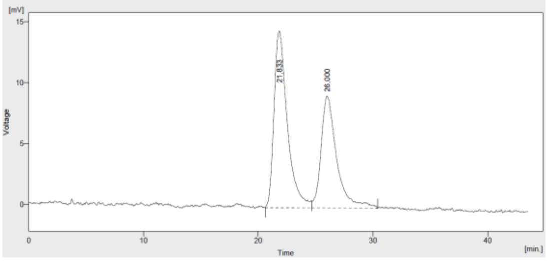		
N°	Retention time (min)	Relative area (%)	N°	Retention time (min)	Relative area (%)
1	22.5	**49.6**	1	21.8	**57.4**
2	27.2	**50.4**	2	26.0	**42.6**

Ir/(*S*)-SEGPHOS complex-catalyzed reaction(Table 2, Entry 2).
The same conditions as above, but $[Ir(COD)_2]BF_4$ was used.

Ni/L* complex-catalyzed reactions (Table 2, Entry 3-5).
A dry microwave vial was charged with Ni(COD)(QD) (0.01 mmol, 3.3 mg) and corresponding chiral ligand (0.02 mmol) in dry toluene (2 mL) under argon atmosphere. Afterwards, the corresponding triynediol **1** (0.1 mmol, 70 mg) was added under argon atmosphere. The reaction

[2] I. Thiel, M. Horstmann, P. Jungk, S. Keller, F. Fisher, H.-J. Drexler, D. Heller, M. Hapke, *Chem. Eur. J.*, **2017**, *23*, 17048-17057.

was stirred at 80 °C for 24 hours. Then, the reaction mixture was concentrated under reduced pressure. The crude diols were directly oxidized to the corresponding diketone **2** without any further purification. To a solution of the crude diols in dry CH_2Cl_2 (5 mL) under argon atmosphere, pyridinium chlorochromate (0.3 mmol, 65 mg) and Celite® (65 mg) were added. The resulting mixture was stirred at 25 °C for 3 hours. Afterwards, the reaction mixture was filtered through a pad of 1:4 silica gel/Celite®. Then, the pad was washed using CH_2Cl_2 and the filtrate was concentrated under reduced pressure. Column chromatography of the residue on silica gel provided products **2**.

The use of (*R*)-QUINAP ligand (Table 2, Entry 3):

HPLC analysis: 44:56 e.r. (column CHIRALPAK IB (Heptane/*i*-PrOH = 85/15, flow rate 0.7 mL/min, UV 238 nm, t_{min} = 10.9 min; t_{major} = 12.6 min).

<table>
<tr><td colspan="3">Racemic</td><td colspan="3">44:56 e.r</td></tr>
<tr><td colspan="3">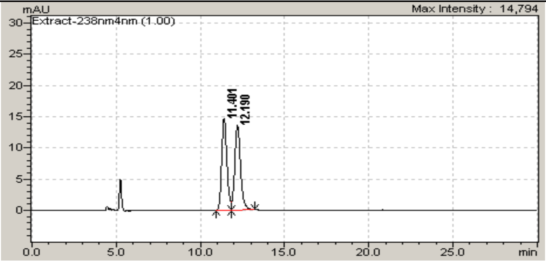
</td><td colspan="3">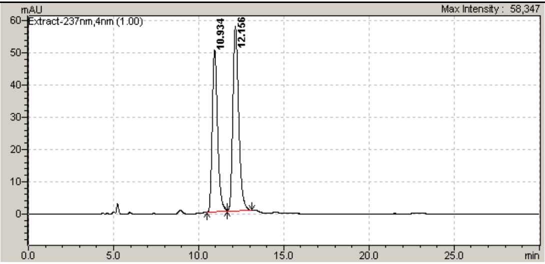
</td></tr>
<tr><td>N°</td><td>Retention time (min)</td><td>Relative area (%)</td><td>N°</td><td>Retention time (min)</td><td>Relative area (%)</td></tr>
<tr><td>1</td><td>11.4</td><td>49.2</td><td>1</td><td>10.9</td><td>44.3</td></tr>
<tr><td>2</td><td>12.2</td><td>50.8</td><td>2</td><td>12.6</td><td>55.7</td></tr>
</table>

The use of (*R*,*R*)-DIOP ligand (Table 2, Entry 4):

HPLC analysis: 50.5:49.5 e.r. (column CHIRALPAK IB (Heptane/*i*-PrOH = 85/15, flow rate 0.7 mL/min, UV 238 nm, t_{major} = 11.2 min; t_{min} = 12.5 min).

<table>
<tr><td colspan="3">Racemic</td><td colspan="3">50.5:49.5 e.r</td></tr>
<tr><td colspan="3">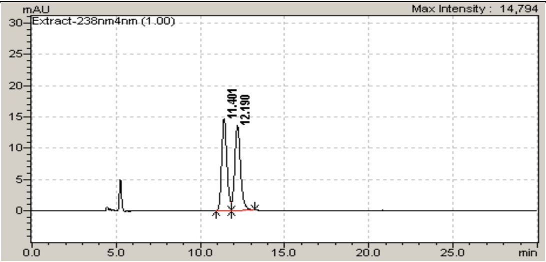
</td><td colspan="3">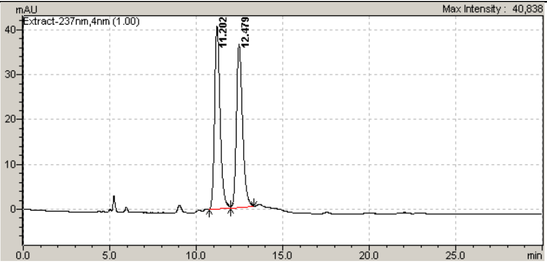
</td></tr>
<tr><td>N°</td><td>Retention time (min)</td><td>Relative area (%)</td><td>N°</td><td>Retention time (min)</td><td>Relative area (%)</td></tr>
<tr><td>1</td><td>11.4</td><td>49.2</td><td>1</td><td>11.2</td><td>50.5</td></tr>
<tr><td>2</td><td>12.5</td><td>50.8</td><td>2</td><td>12.5</td><td>49.5</td></tr>
</table>

Co/L* complex-catalyzed reactions (Table 2, Entries 6-8).

A Schlenk tube was charged with $CoBr_2$ (0.0125 mmol, 2.7 mg), the respective chiral ligand (0.0125 mmol), Zn (0.025 mmol, 1.6 mg) as well as ZnI_2 (0.025 mmol, 8.0 mg) and THF (3 mL) was added. The solution was heated for 1 h to 60 °C before being cooled to room temperature gain with subsequent addition of triynediol **1** (0.05 mmol, 34.9 mg). Afterwards, the reaction solution was heated to the given temperature and for the given time (Table S1). Then, the crude diols were directly oxidized to the corresponding ketones without any further purification. To

a solution of the crude diols in dry CH_2Cl_2 (5 mL) under argon atmosphere, pyridinium chlorochromate (0.15 mmol, 32.6 mg) and Celite® (32.6 mg) were added. The resulting mixture was stirred at 25 °C for 3 h. Afterwards, the reaction mixture was filtered through a pad of a 1:4 mixture of silica gel/Celite®. Then, the pad was washed using CH_2Cl_2 and the filtrate was concentrated under reduced pressure. Column chromatography of the residue on silica gel (6/1/1 v/v/v, hexanes/EtOAc/CH_2Cl_2) provided compound **2** and/or **3** like given in Table S1. The formed compounds **2** showed no enantiomeric excess.

Table S1: Yields of the applied chiral cobalt-catalyzed reactions and selected investigated parameters of helicene **3** formation.

Entry	Ligand[a]	T (°C)	t (h)	Yield **2** (%)	Yield **3** (%)
1	(*S*)-1-(Diphenylphosphino)-2-[(*S*)-4-isopropyloxazolin-2-yl] ferrocene	60	48	-	-[c]
2	(*R*)-QUINAP	60	48	-	-
3	(*R*)-QUINAP	80	48	-	-
4	(*R*)-QUINAP	80	96	6	-
5	(*R*)-QUINAP[b]	80	48	-[c]	16
6	(*R*)-QUINAP	120	16	33	18
7	(R_a,*R*)-*O*-PINAP	120	48	-	89
8	(R_a,*S*)-Ph-Bn-SIPHOS	120	48	26	50
9	(R_a,*S*)-Ph-Bn-SIPHOS[b]	60	48	11[c]	33
10	(*S*)-H_8-BINAP[b]	60	48	-	-[c]
11	(*R*,*R*)--DIOP[b]	60	48	-	-[c]

[a] 25 mol% cobalt(II) bromide, 25 mol% ligand, 50 mol% zinc and 50 mol% of zinc(II) iodide were used unless mentioned.
[b] 50 mol% cobalt(II) bromide, 50 mol% ligand, 100 mol% zinc and 100 mol% of zinc(II) iodide were used.
[c] The formed product was not separable from side-product(s).

5 Mechanistic investigations

5.1 DFT Calculations

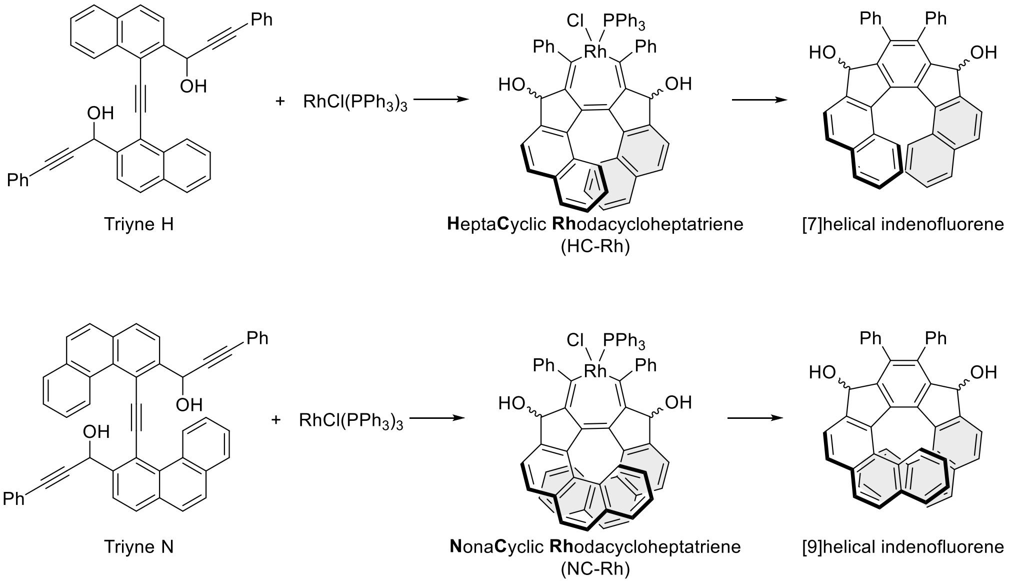

Scheme S1.

Table S2. Decrease of total energy achieved by the formation of intermediate isomers after the reaction between their respective organic precursors and $Rh(PPh_3)_2Cl$.

	ΔE_{TOT}
HC-Rh	-327.0
NC-Rh	-306.7

Table S3. Stabilization energy of adduct formation between the organic precursors and $RhCl(PPh_3)_3$.

	ΔE_{TOT}
HC-Rh	43.7
NC-Rh	5.8

Table S4. Gibbs energies (p = 1 atm and T = 353.15 K) of intermediate isomers with respect to the organic precursors with $Rh(PPh_3)_2Cl$.

	ΔG
HC-Rh	-179.3
NV-Rh	-161.0

5.2 Reaction mixtures analyses

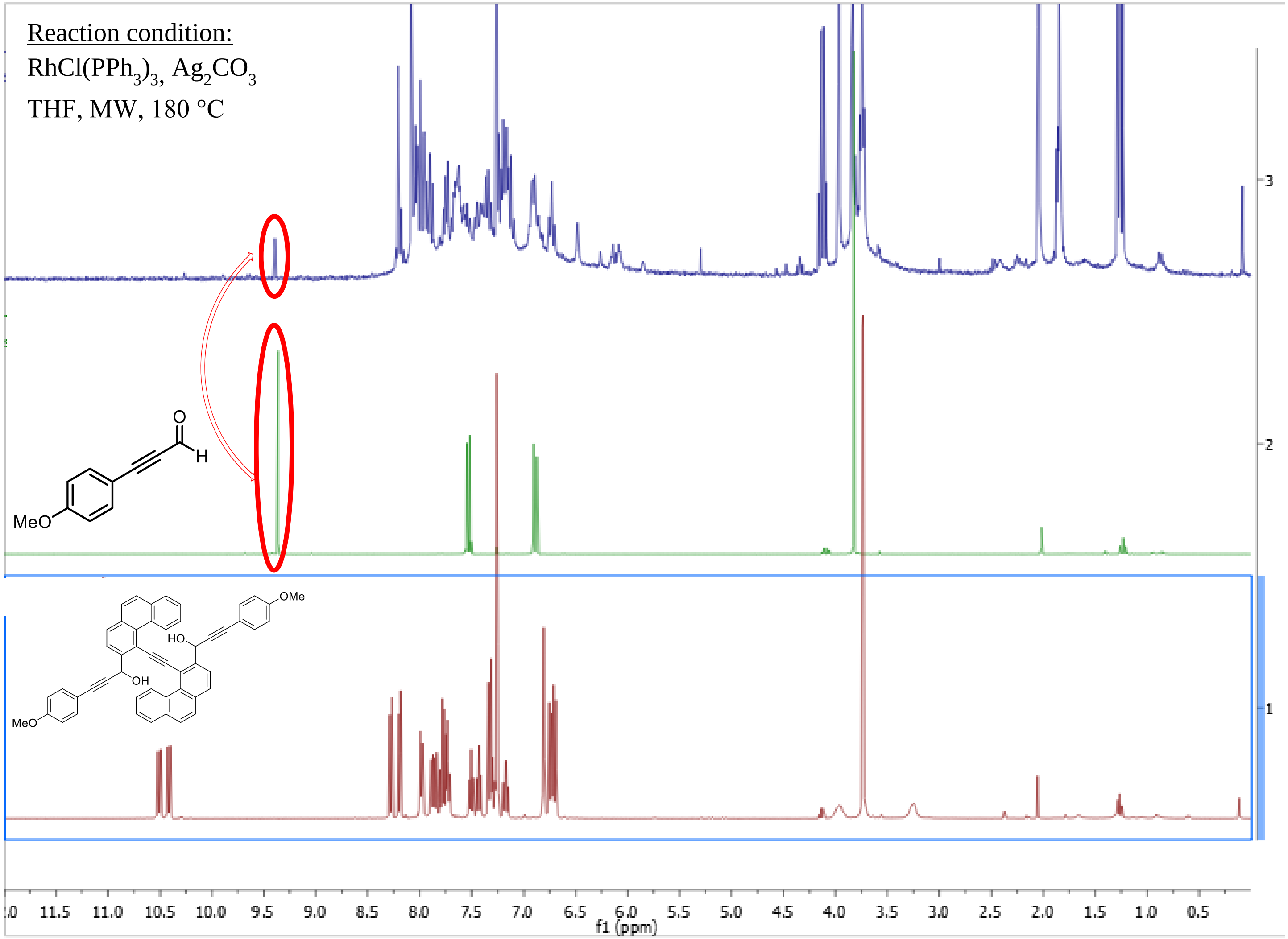

Figure S1. ^{1}H NMR investigations. (a) ^{1}H NMR of the crude mixture from cyclotrimerization performed with our standard condition (the blue spectrum). (b) ^{1}H NMR of 3-(4-methoxyphenyl)propiolaldehyde (green spectra). (c) ^{1}H NMR of the triyne **1** (the red spectrum).

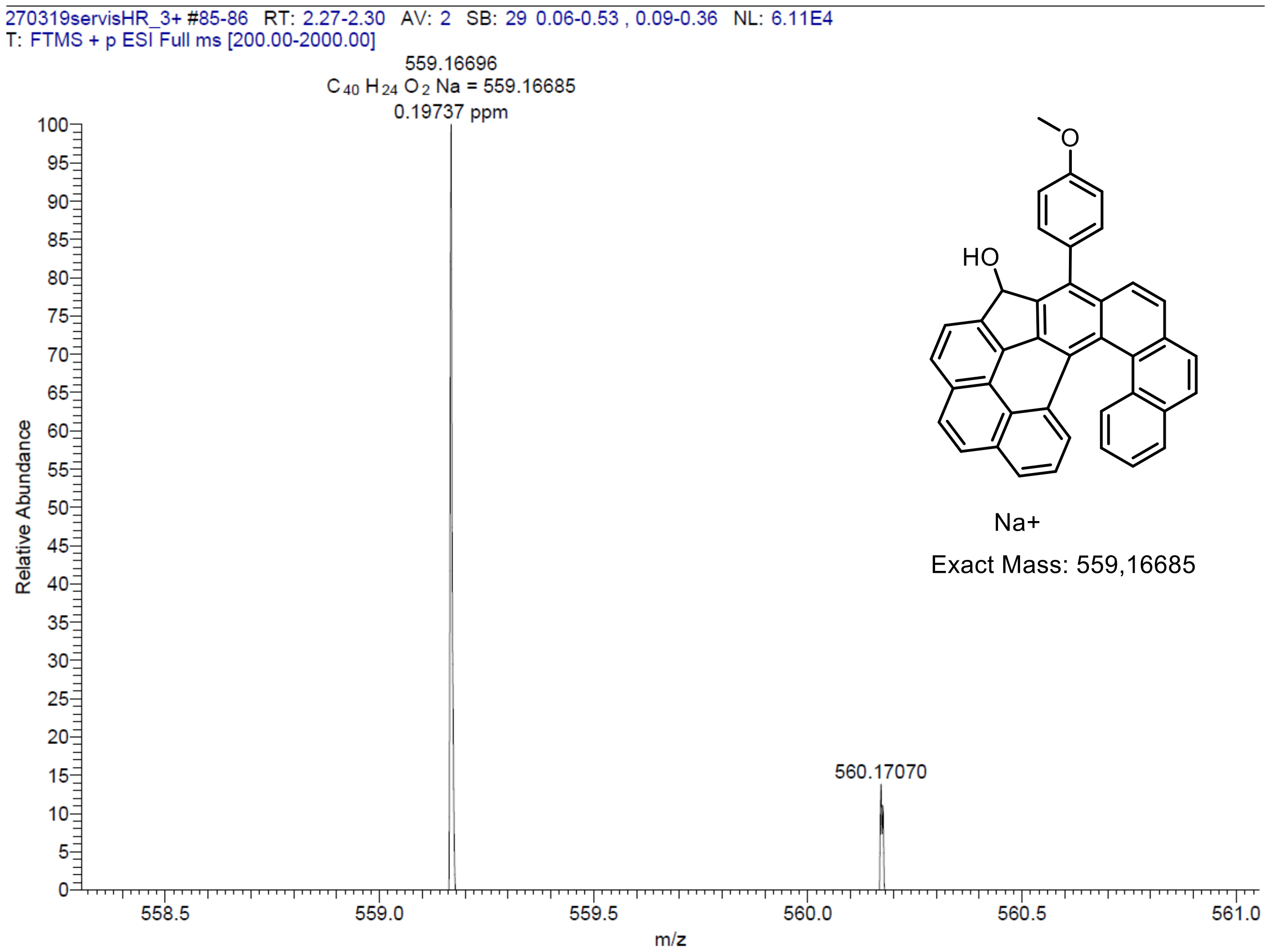

Figure S2. HRMS of alcohol **VI** from obtained by a DDA reaction (reaction condition: $RhCl(PPh_3)_3$, Ag_2CO_3, THF, MW, 170 °C, 1.5 h).

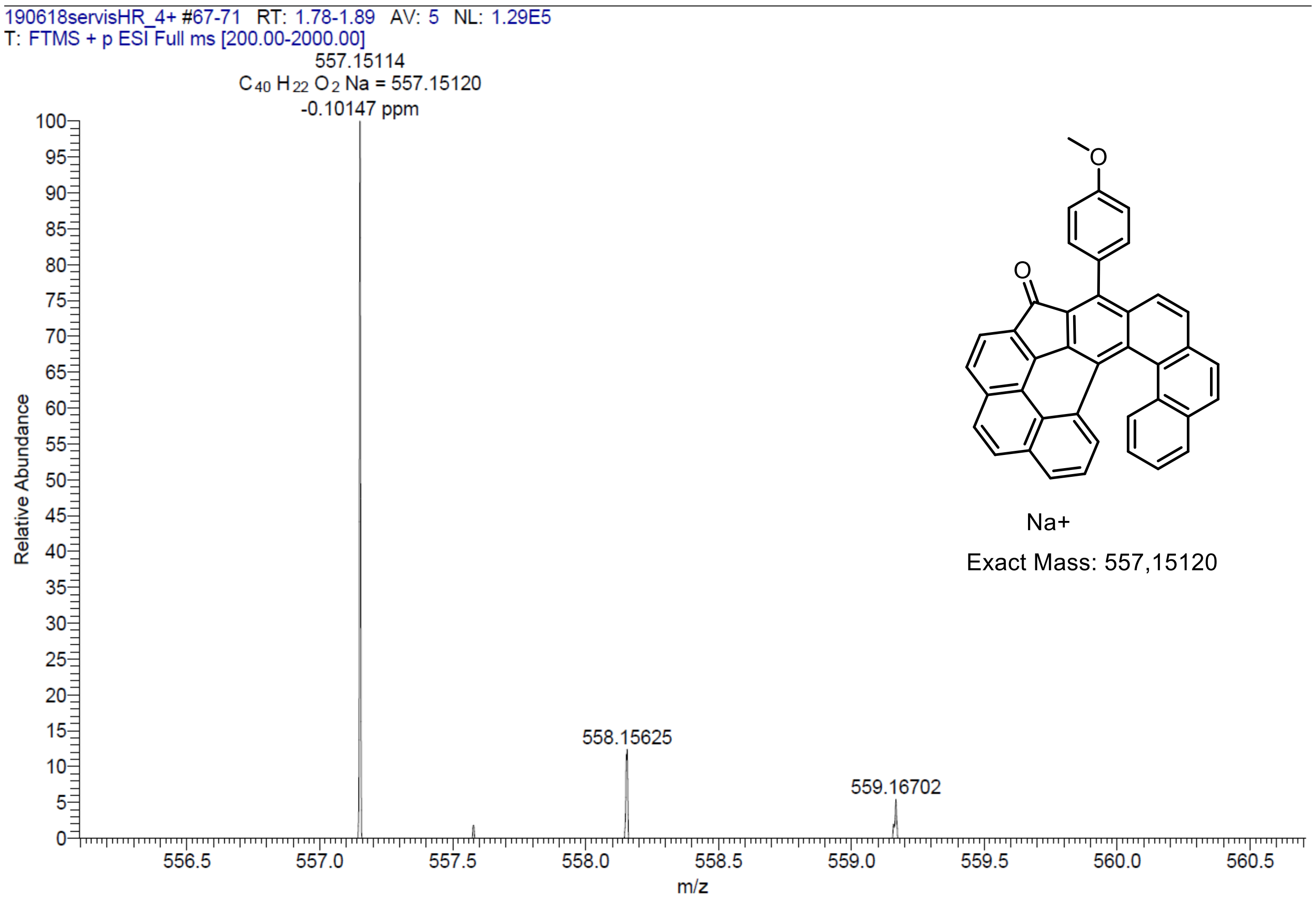

Figure S3. HRMS of compound **3**. (oxidation of alcohol **VI** by using PCC in CH_2Cl_2)

6 Synthesis of spirofluorene derivatives

10',11'-Bis(4-methoxyphenyl)dispiro[fluorene-9,9'-as-indaceno[2,1-c:7,8-c']diphenanthrene-12',9''-fluorene] (4). To a solution of 2-bromobiphenyl (0.6 mmol, 110 μL) in anhydrous THF (6 mL), *n*-BuLi (1.6 M, 0.6 mmol, 0.4 mL) was added dropwise at -78 °C. The resulting solution was stirred for 30 min, followed by the dropwised addition of a solution of **2** (0.1 mmol, 70 mg) in Toluene (7 mL). The reaction mixture was stirred first at -78 °C for 30 min and 16 hours at room temperature. The mixture was quenched with a solution of saturated NH_4Cl and extracted with EtOAc (3 × 20 mL). The combined organic layers were dried over Na_2SO_4, filtered and concentrated under reduced pressure. The alcohol was dissolved in AcOH (25 mL) with 12 M HCl (0.5 mL) and the resulted mixture was stirred under reflux overnight. Chromatography column of the residue on silica gel (6/1 hexanes/EtOAc) provided 28 mg (29%) of the title compound as a yellowish solid.

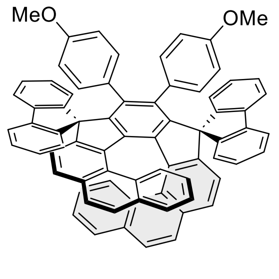

R_f = 0.32 (6/1 hexanes/EtOAc).

Mp > 320 °C.

^{1}H NMR (400 MHz; $CDCl_3$) δ_H 7.86 (d, *J* = 8.8 Hz, 2H), 7.46 (dd, *J* = 6.8, 1.6 Hz, 2H), 7.38-7.24 (m, 12H), 7.21 (d, *J* = 8.7 Hz, 2H), 7.17-7.08 (m, 4H), 7.03-6.87 (m, 6H), 6.72 (d, *J* = 7.9 Hz, 2H), 6.32 (ddd, *J* = 8.3, 7.0, 1.3 Hz, 2H), 5.93-5.87 (m, 4H), 5.85-5.77 (m, 4H), 3.54 (s, 6H).

^{13}C NMR (100 MHz; $CDCl_3$) δ_C 156.6 (2C), 150.0 (2C), 149.2 (2C), 148.3 (2C), 145.3 (2C), 143.0 (2C), 142.0 (2C), 138.5 (2C), 138.0 (2C), 137.7 (2C), 131.3 (2C), 131.2 (2C), 131.0 (2C), 130.3 (2C), 129.3 (2C), 128.5 (2C), 128.2 (2C), 128.0 (2C), 127.48 (2C), 127.47 (2C), 127.22 (2C), 127.17 (2C), 126.7 (2C), 126.3 (2C), 125.8 (2C), 125.2 (2C), 124.7 (2C), 124.3 (2C), 123.8 (2C), 121.6 (2C), 120.4 (2C), 120.1 (2C), 119.9 (2C), 111.6 (2C), 111.4 (2C), 66.7 (2C), 55.1 (2C).

IR (KBr) ν_{max} 3060, 3045, 3014, 2991, 2962, 2927, 2854, 2833, 1734, 1612, 1516, 1473, 1446, 1284, 1244, 1176, 1105, 1039, 833, 802, 793, 752, 737 cm^{-1}.

HRMS (APCI): m/z calcd for $C_{74}H_{47}O_2$ [(M+H)$^+$]: 967.35706, found: 967.35695.

6-(4-Methoxyphenyl)spiro[benzo[*no*]indeno[2,1,7,6-*ghij*]naphtho[1,2-*a*]tetraphene-5,9'-fluorene] (5). To a solution of 2-bromobiphenyl (0.3 mmol, 52 μL) in anhydrous THF (3 mL), *n*-BuLi (1.6 M, 0.3 mmol, 0.19 mL) was added at -78 °C. The resulting solution was stirred for 30 min, followed by the addition of a solution of **3** (0.13 mmol, 70 mg) in THF (6 mL). The reaction mixture was stirred first at -78 °C for 30 min and 3 hours at room temperature. The mixture was quenched with a solution of saturated NH_4Cl and extracted with EtOAc (3 × 20 mL). The combined organic layers were dried over Na_2SO_4, filtered and concentrated under reduced pressure. The alcohol was dissolved in AcOH (5 mL) with 12 M HCl (0.5 mL) and the resulted mixture was stirred under reflux for 2 hours. Chromatography column of the residue on silica gel (6/1 hexanes/EtOAc) provided 61 mg (70%) of the title compound as a yellowish solid.

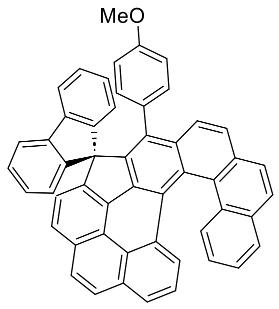

R_f = 0.43 (6/1 hexanes/EtOAc).

Mp = 176-180 °C.
^{1}H NMR (400 MHz; $CDCl_3$) δ_H 8.18 (d, *J* = 8.1 Hz, 1H), 8.14-7.94 (m, 7H), 7.87 (dd, *J* = 8.1, 0.9 Hz, 1H), 7.78 (d, *J* = 8.6 Hz, 1H), 7.72 (d, *J* = 8.6 Hz, 1H), 7.63 (d, *J* = 7.7 Hz, 1H), 7.52 (d, *J* = 7.7 Hz, 1H), 7.43 (d, *J* = 7.8 Hz, 1H), 7.40-7.21 (m, 4H), 7.12 (td, *J* = 7.4, 1.1 Hz, 1H), 7.04 (d, *J* = 7.6 Hz, 1H), 6.96 (td, *J* = 7.5, 1.1 Hz, 1H), 6.83-6.79 (m, 1H), 6.55 (d, *J* = 7.6 Hz, 1H), 6.48-6.37 (m, 3H), 6.24-6.21 (m, 1H), 3.79 (s, 3H).
^{13}C NMR (100 MHz; $CDCl_3$) δ_C 158.1, 147.9, 145.5, 144.3, 142.4, 142.2, 137.4, 136.3, 135.8, 135.7, 131.9, 131.73, 131.70, 130.9, 130.8, 130.6, 129.8, 129.6, 129.2, 128.6, 128.3, 128.2, 127.80, 127.77, 127.53, 127.51, 127.4, 126.9, 126.7, 126.5, 126.1, 126.0, 125.84, 125.76, 125.7, 125.6, 125.5, 125.14, 125.07, 124.9, 124.5, 124.2, 123.6, 122.0, 121.3, 120.1, 119.9, 112.8, 112.7, 68.5, 55.5.
IR (KBr) ν_{max} 3387, 3049, 2966, 2937, 2836, 2187, 1604, 1506, 1419, 1293, 1251, 1174, 1031, 954, 836, 748 cm^{-1}.
HRMS (APCI): *m/z* calcd for $C_{52}H_{31}O$ [$(M+H)^+$]: 671.23694, found: 671.23751.

7 X-Ray diffraction analysis

X-ray experiments for single crystal structure determination were performed were on Bruker D8 VENTURE Kappa Duo PHOTON100 by IμS micro-focus sealed. Crystals were cooled to low temperature maintained by Oxford Cryostream Cooler**.** The multi-scan absorption corrections were carried on. The structures were solved by direct methods (XT[3]) and refined by full matrix least squares based on F^2 (SHELXL2018[4]). The hydrogen atoms on carbon were fixed into idealised positions (riding model) and assigned temperature factors either $H_{iso}(H) = 1.2\ U_{eq}$(pivot atom) or $H_{iso}(H) = 1.5\ U_{eq}$ (pivot atom) for methyl moiety.

The solvent molecules were found in unit cells of all samples. The real structure of **2** (tim_214_kotora_5) is complicated by non-merohedric twinning, its refinement resulted in domain ration 0.57_0.43.

[3] SHELXT: Sheldrick, G.M. (2015). Acta Cryst. A71, 3-8.
[4] SHELXL: Sheldrick, G.M. (2015). Acta Cryst. C71, 3-8.

Table S4. Crystal data, data collection, and refinement parameters for **??????**

**Compound**	**2** (tim_214_kotora_5)	**3** (tim_step7)
CCDC	2261993	2261994
Formula	$C_{50}H_{30}O_4·CH_2Cl_2$	$C_{40}H_{22}O_2·2(CH_2Cl_2)$
M.w.	779.66	704.43
Crystal system	Triclinic	Triclinic
Space group	*P*-1 (No.2)	*P*-1 (No.2)
a [Å]	9.2652 (4)	10.8565 (6)
b [Å]	12.7594 (6)	11.0629 (6)
c [Å]	16.0092 (7)	14.7305 (7)
α [°]	93.041 (2)	104.632 (2)
β [°]	95.280 (2)	97.400 (2)
γ [°]	95.348 (2)	108.757 (2)
Z	2	2
$V [Å^3]$	1872.72 (15)	1577.74 (15)
Temperature [K]	120	120
Wavelength [$Å^3$]	1.54178	0.71073
D_x [g cm^{-3}]	1.383	1.483
Crystal size [mm]	0.46 × 0.10 × 0.05	0.34 × 0.26 × 0.14
Crystal color, shape	Bar, orange	Prism, orange
μ [mm^{-1}]	1.96	0.42
T_{min}, T_{max}	0.76, 0.91	0.89, 0.94
Measured reflections	47982	33806
Independent diffractions (R_{int}^a)	7039, (0.036)	7280, (0.023)
Observed diffract. [I>2σ(I)]	6563	6419
No. of parameters	517	343
R^b	0.043	0.042
$wR(F^2)$ for all data	0.115	0.113
GOF^c	1.04	1.03
Residual electron density [e/Å^3]	0.51, −0.82	1.02, −0.99

$^aR_{int} = \Sigma|F_o^2 - F_{o,mean}^2|/\Sigma F_o^2$;

$^bR(F) = \Sigma||F_o| - |F_c||/\Sigma|F_o|$; $wR(F^2) = [\Sigma(w(F_o^2 - F_c^2)^2)/(\Sigma w(F_o^2)^2)]^{½}$;

^{c}GOF $= [\Sigma(w(F_o^2 - F_c^2)^2)/(N_{diffrs} - N_{params})]^{½}$

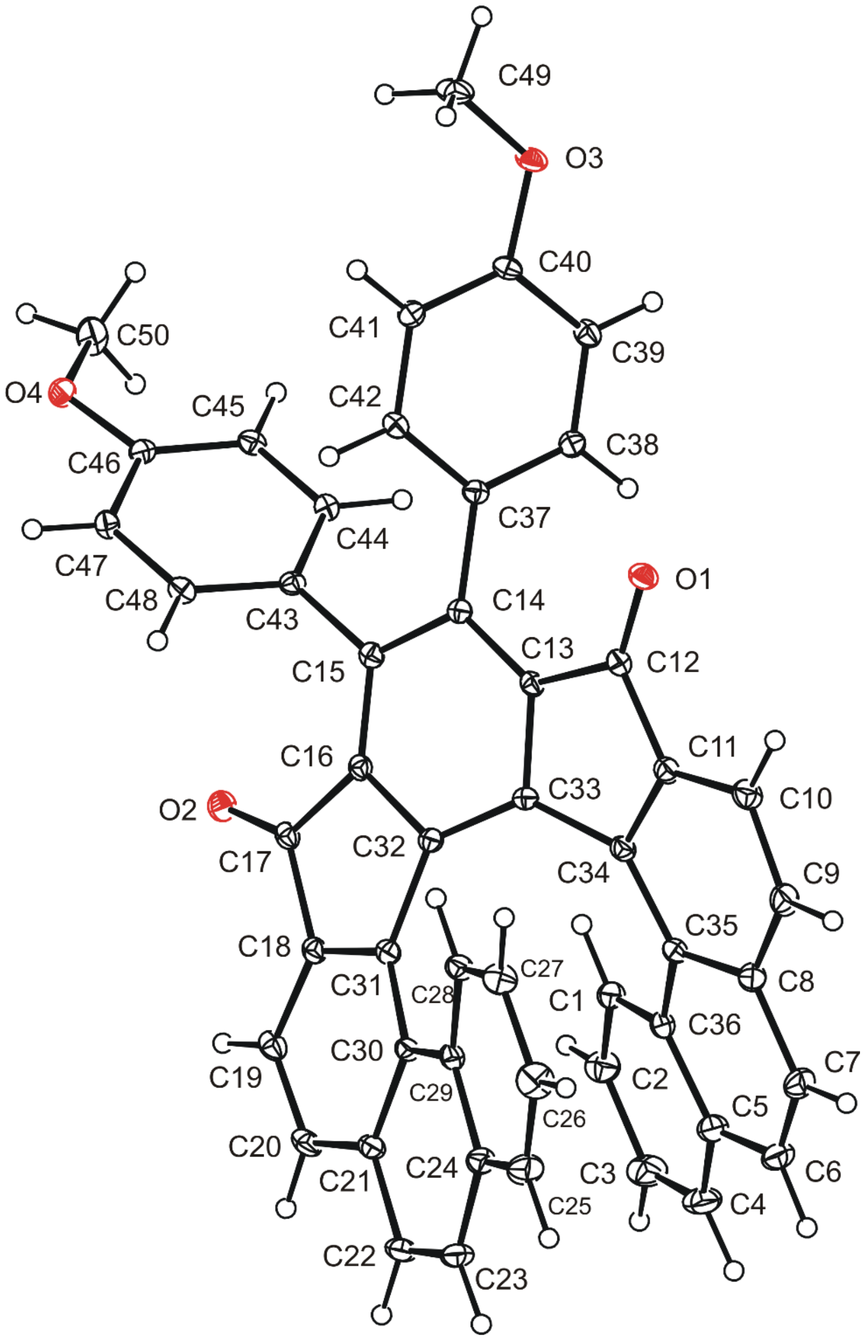

Figure S4. View of **2** with atom numbering schema. The displacement ellipsoids are drawn on 30% probability level.

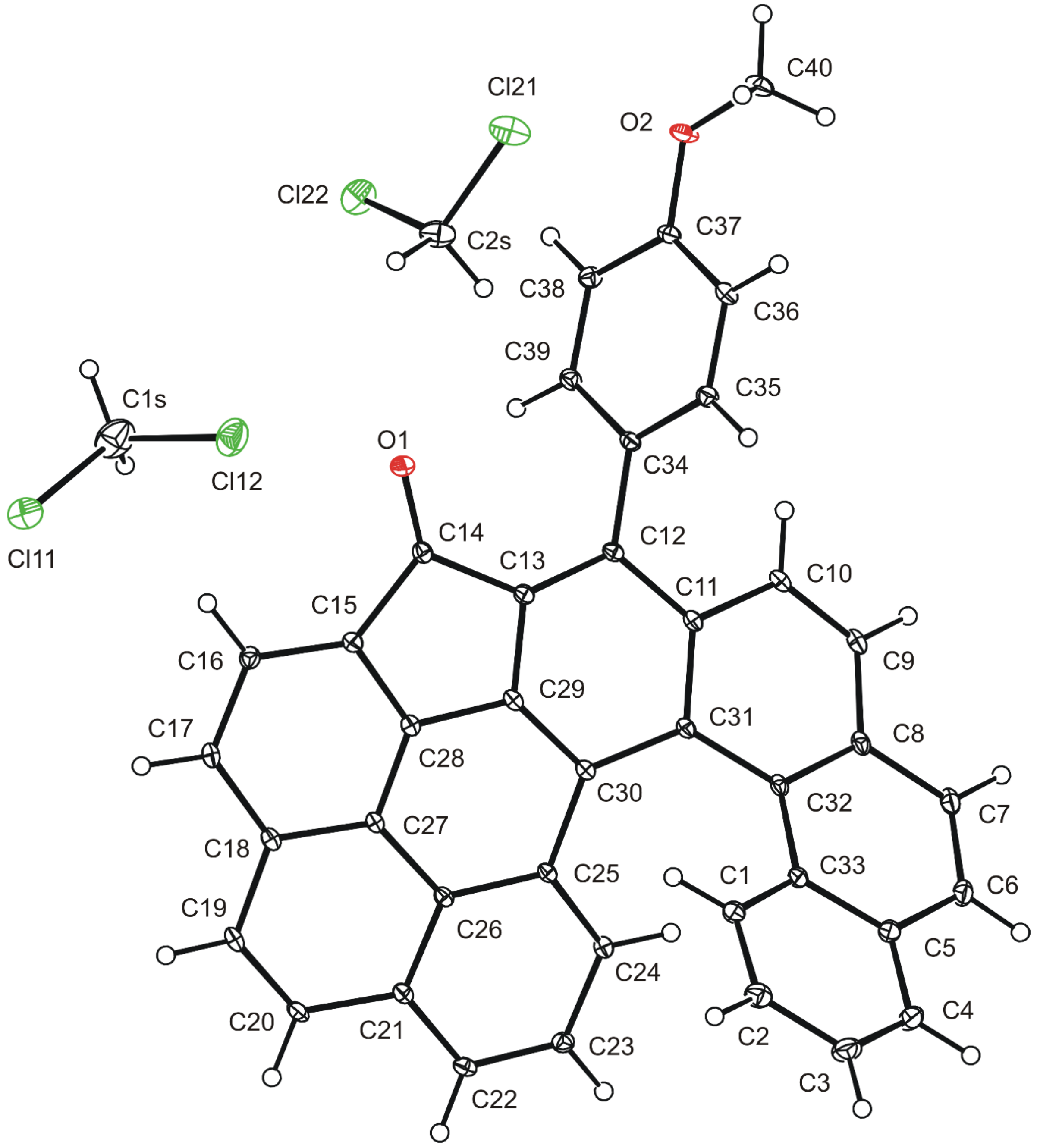

Fig ure S5. View of **3** with atom numbering schema. The displacement ellipsoids are drawn on 30% probability level.

8 Copies of ¹H and ¹³C NMR spectra

4-Bromo-1,2-dihydrophenanthrene-3-carbaldehyde (S1).

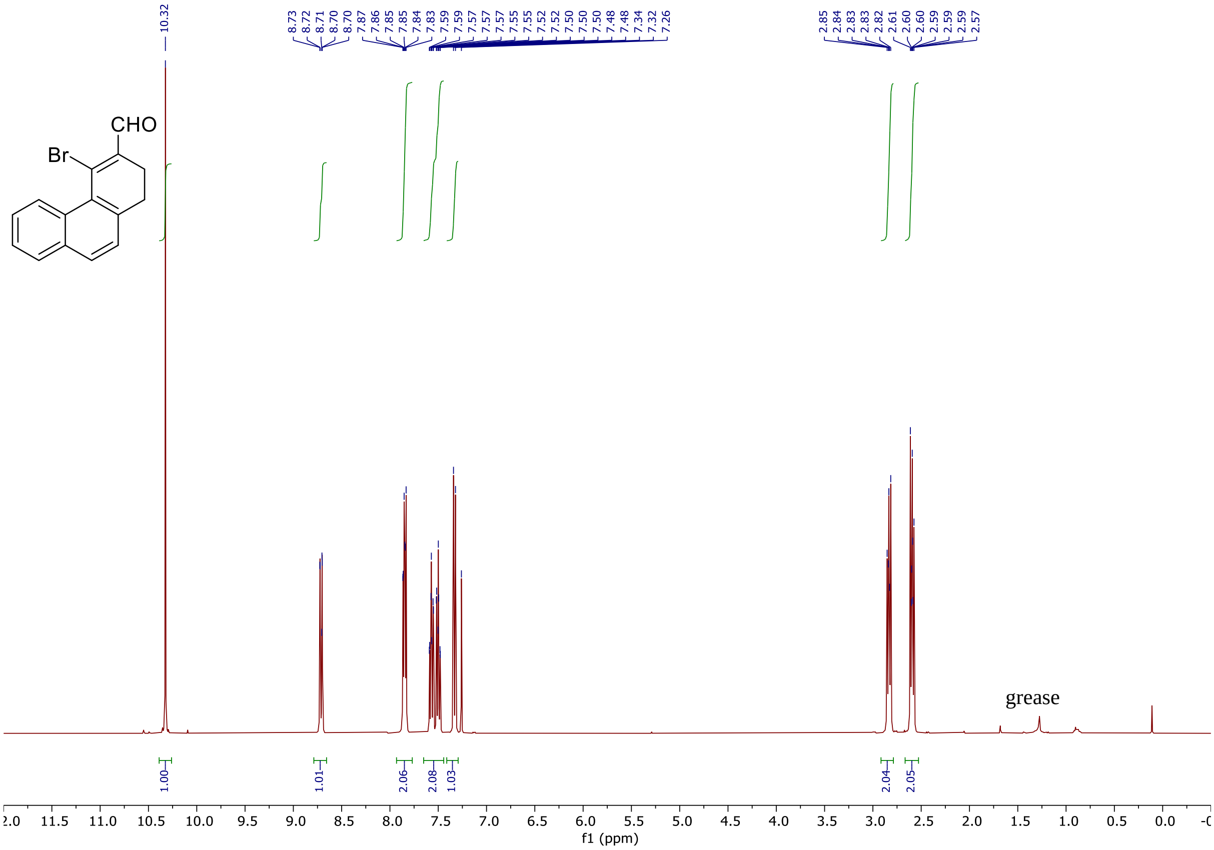

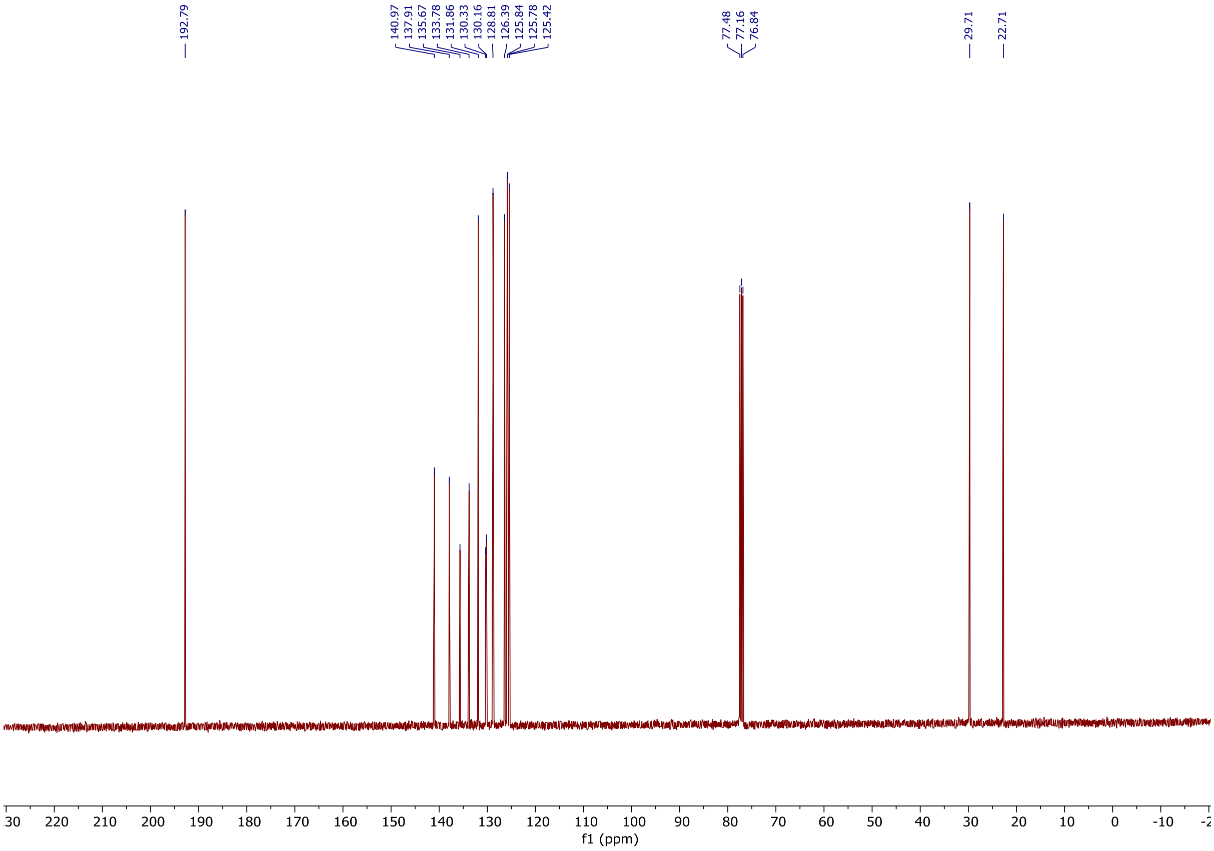

4-Bromophenanthrene-3-carbaldehyde (S2).

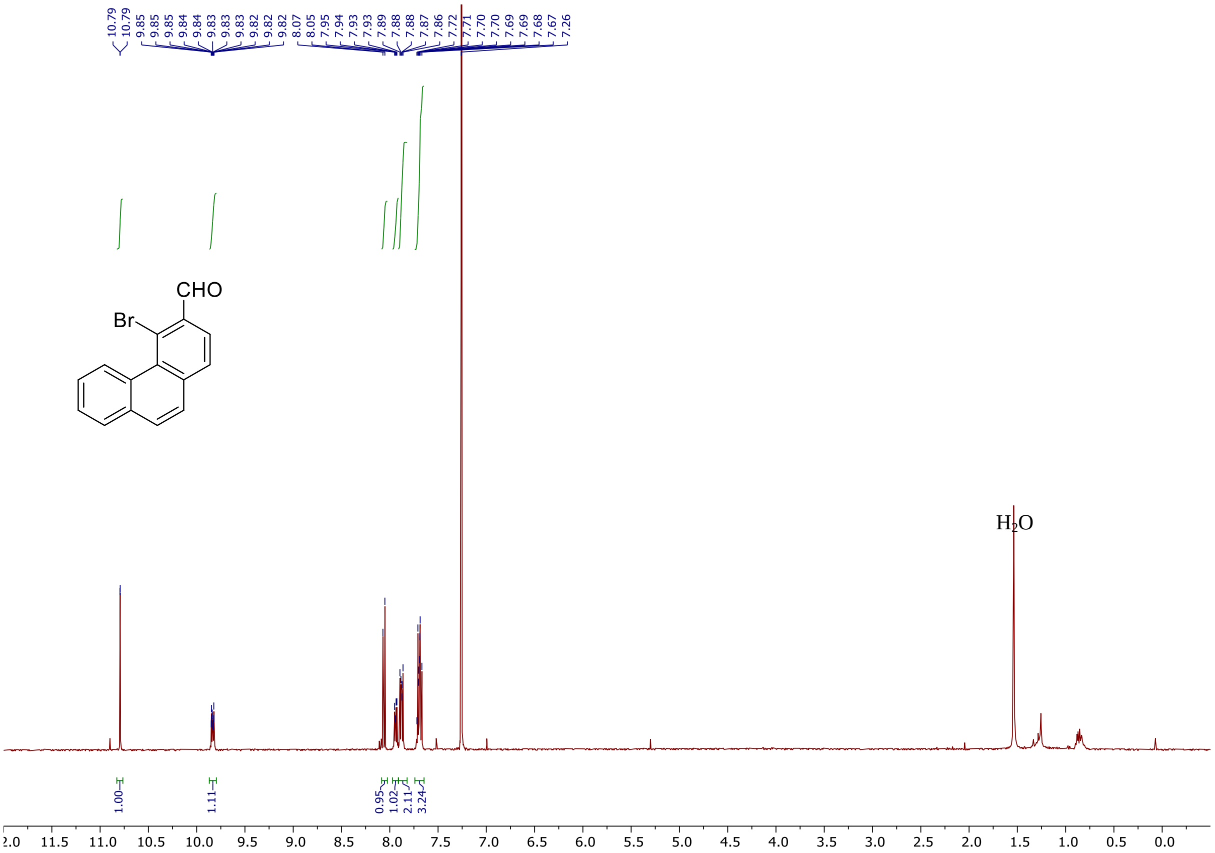

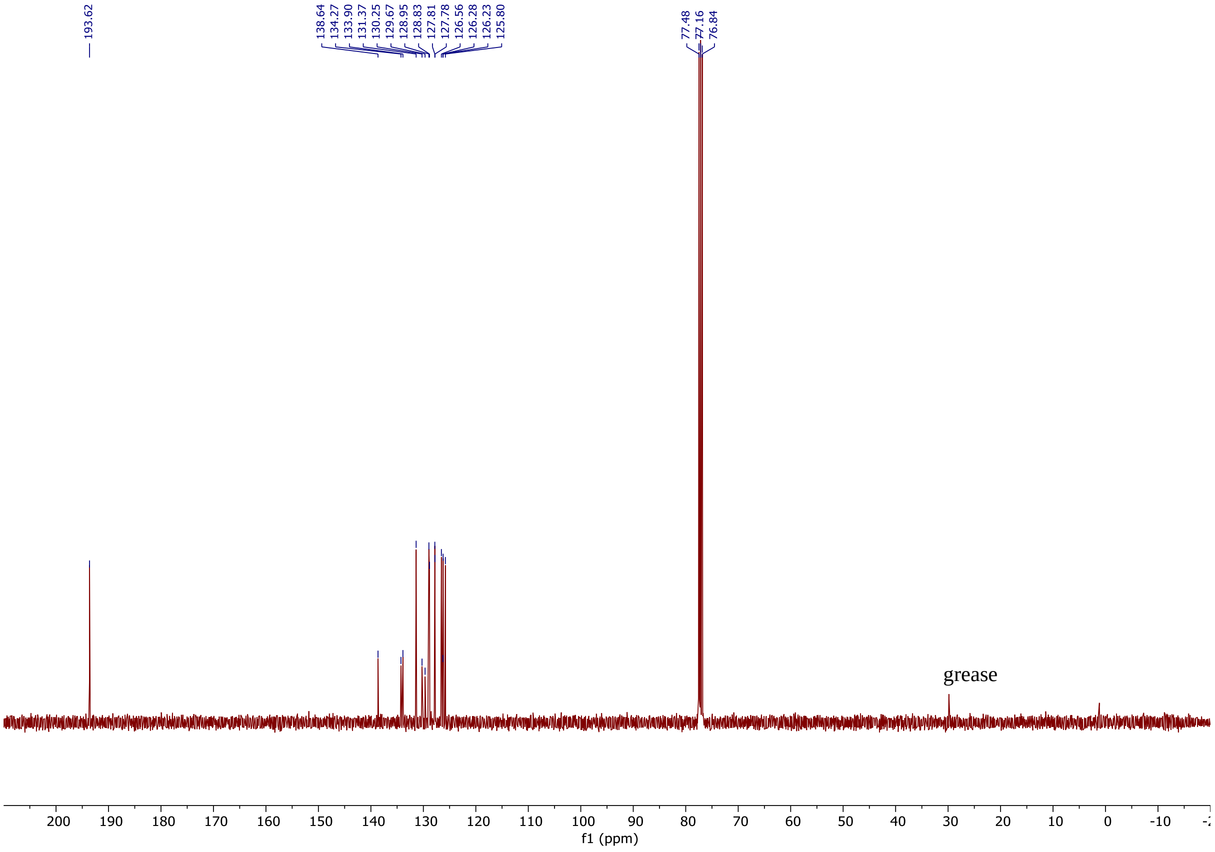

4-Ethynylphenanthrene-3-carbaldehyde (S3).

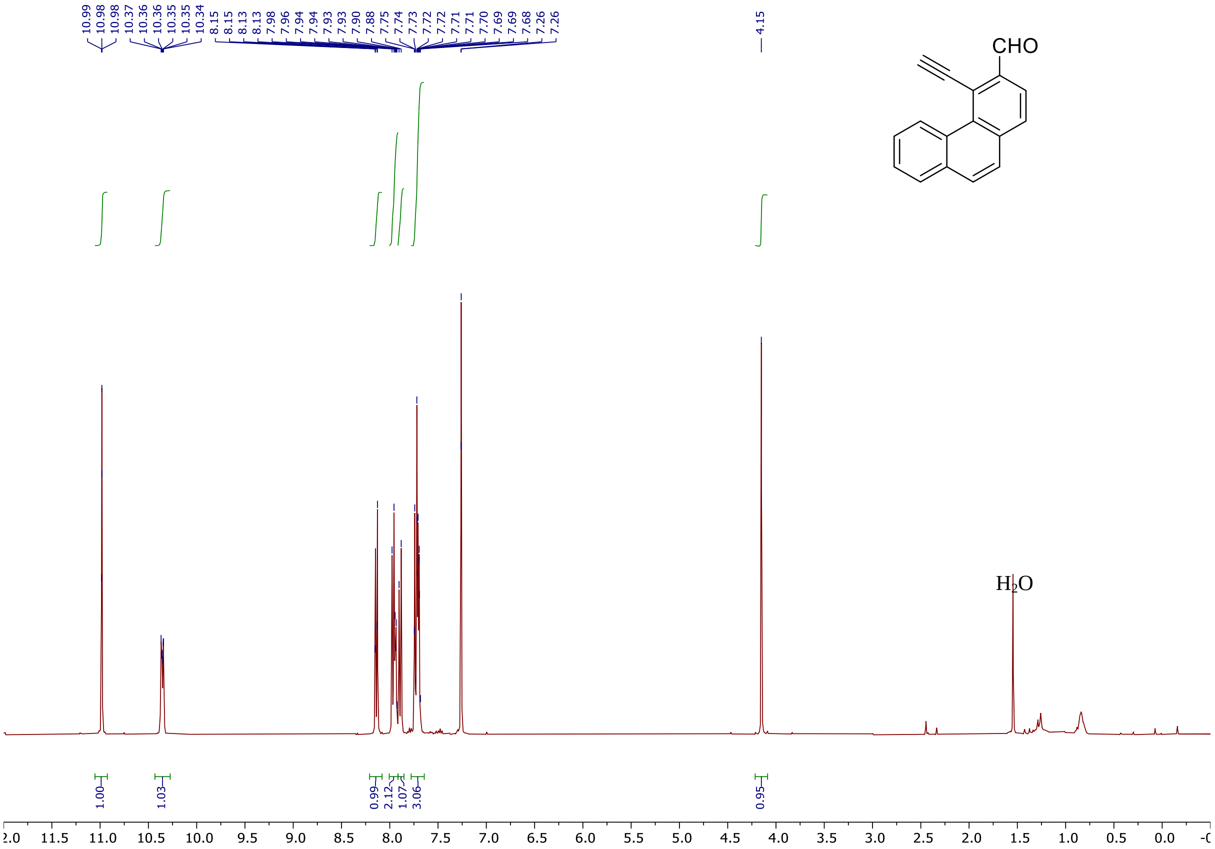

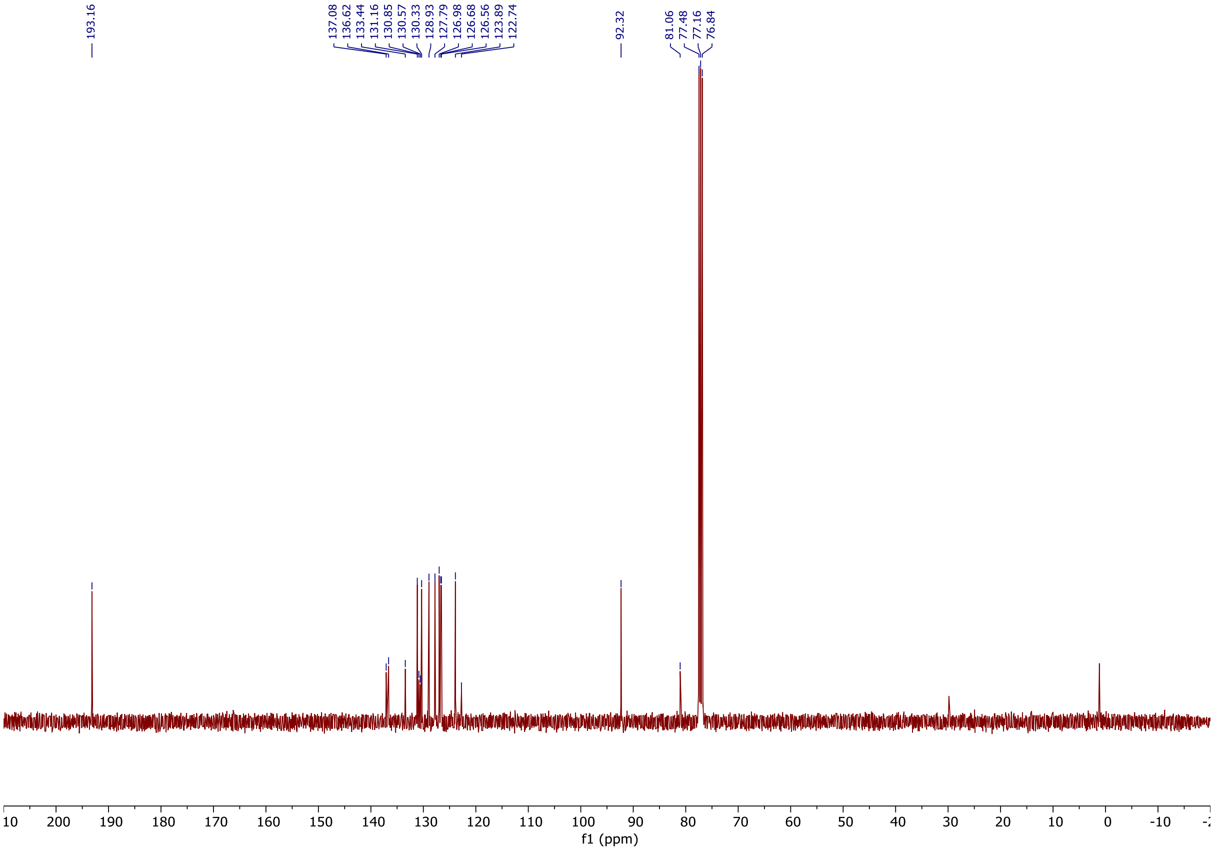

4,4'-(ethyne-1,2-diyl)bis(phenanthrene-3-carbaldehyde) (S4).

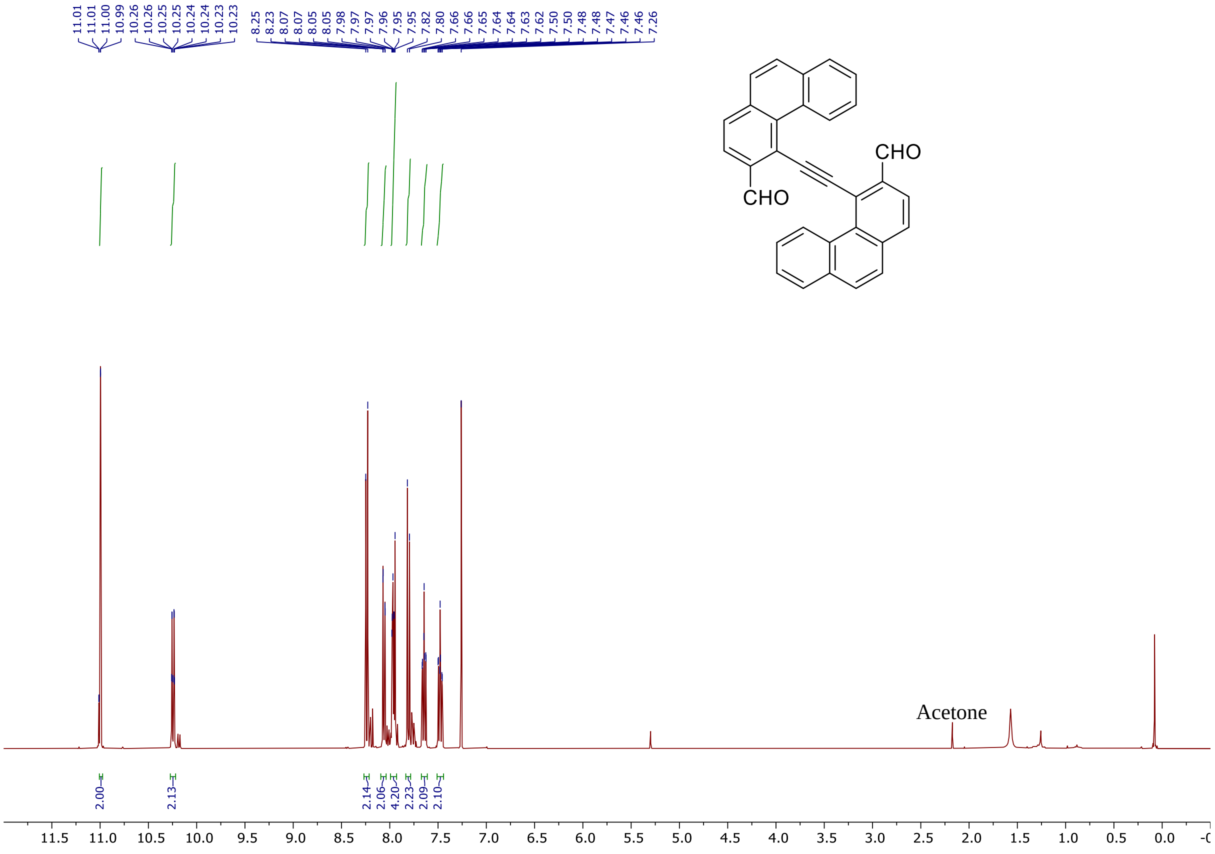

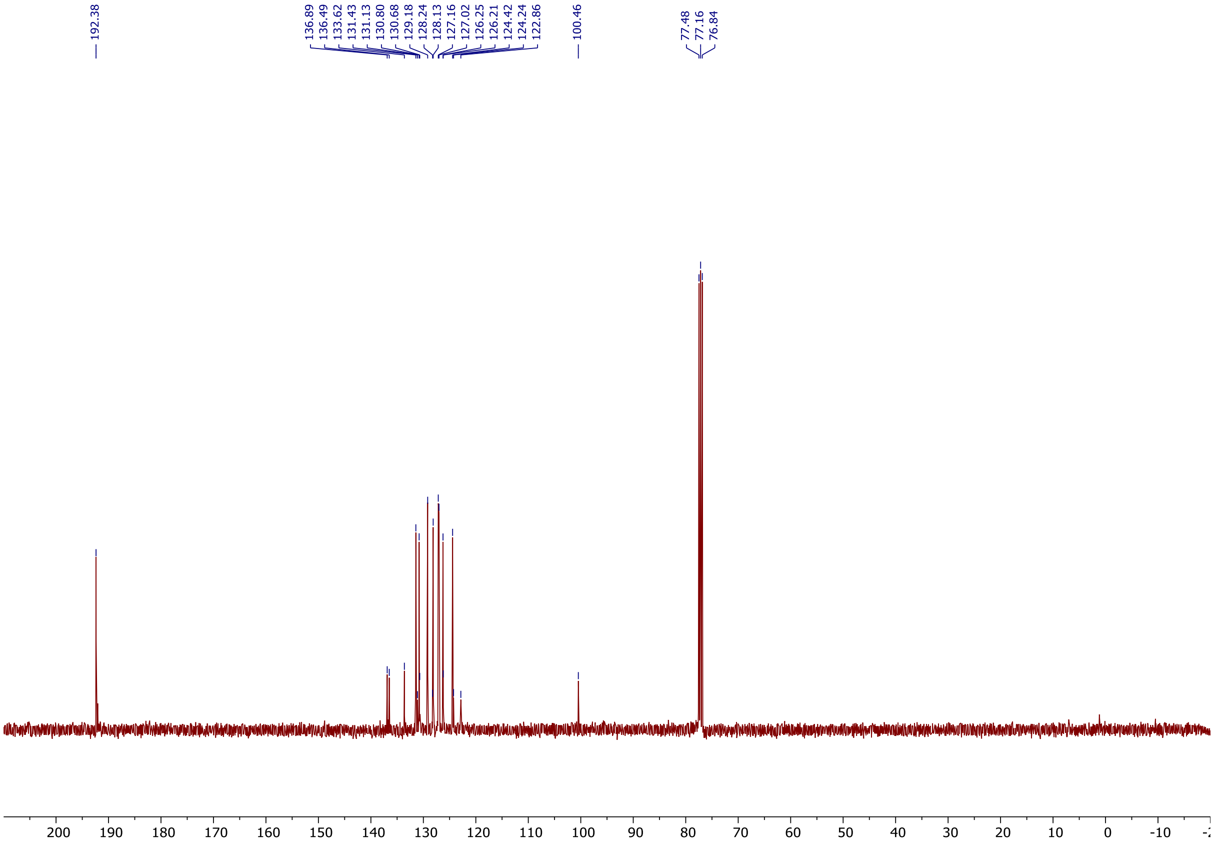

1,1'-(ethyne-1,2-diylbis(phenanthrene-4,3-diyl))bis(3-(4-methoxyphenyl)prop-2-yn-1-ol). (1).

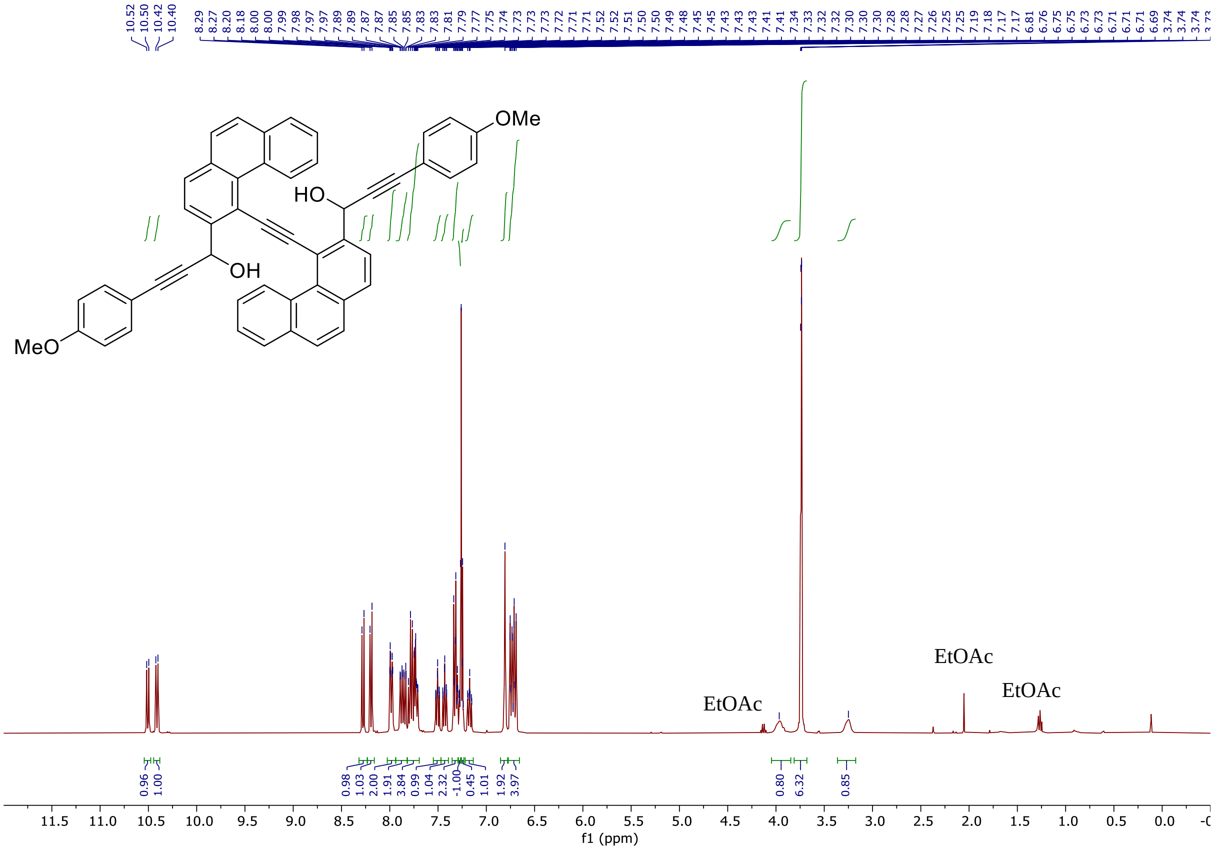

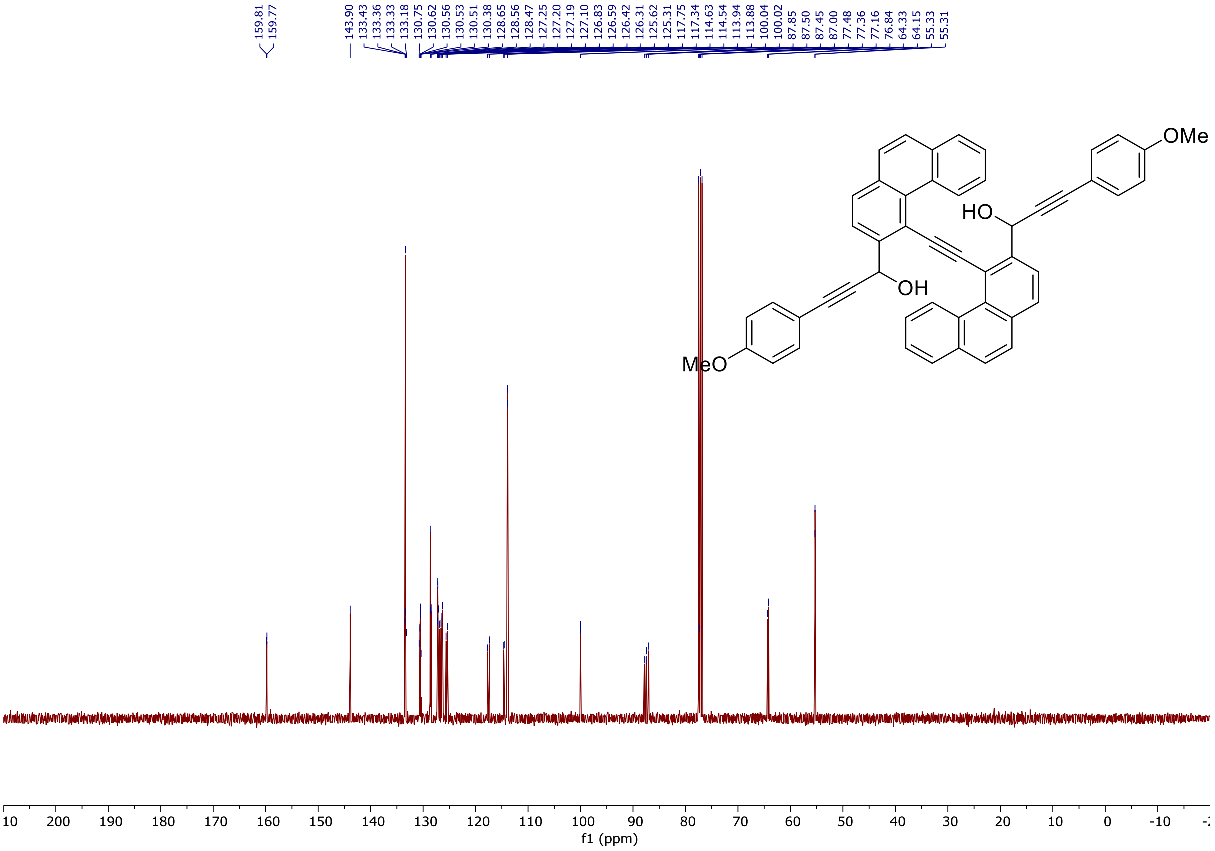

10,11-bis(4-methoxyphenyl)-as-indaceno[2,1-*c*:7,8-*c'*]diphenanthrene-9,12-dione (2).

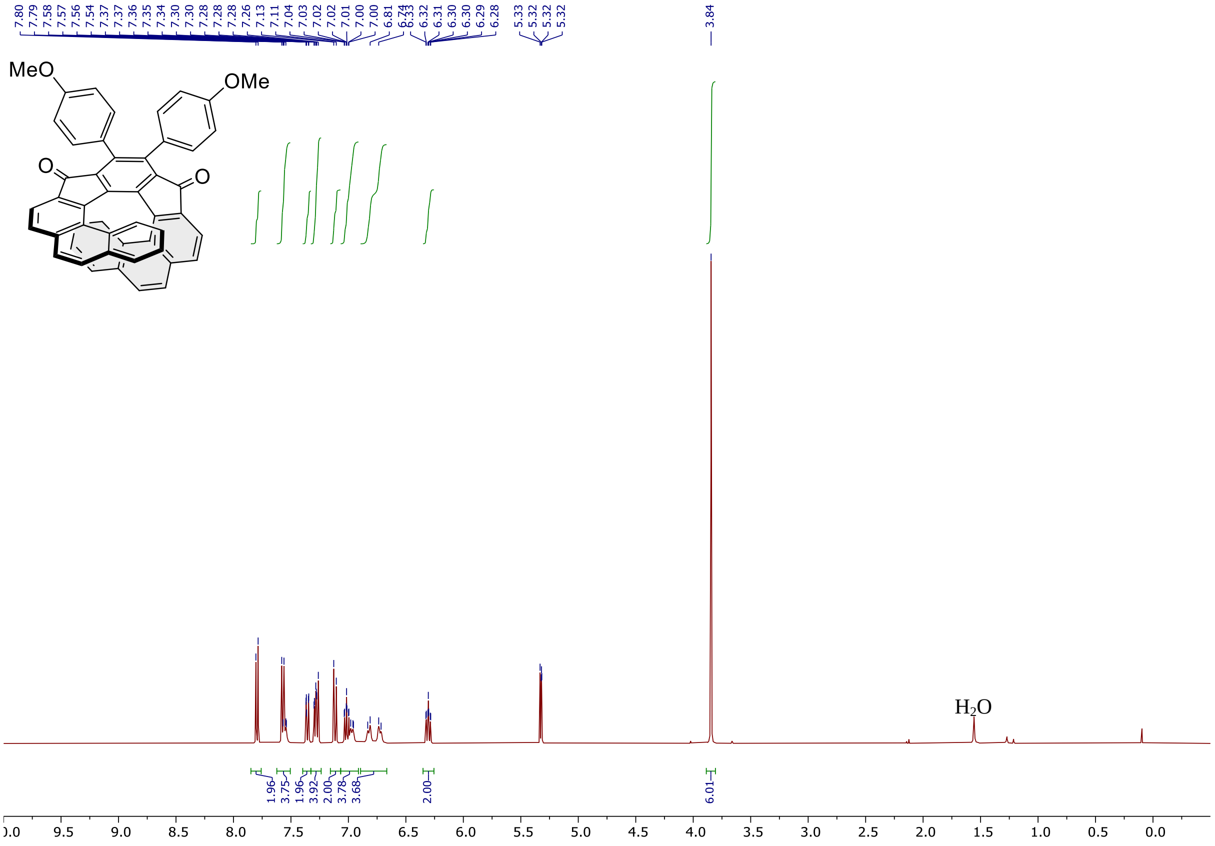

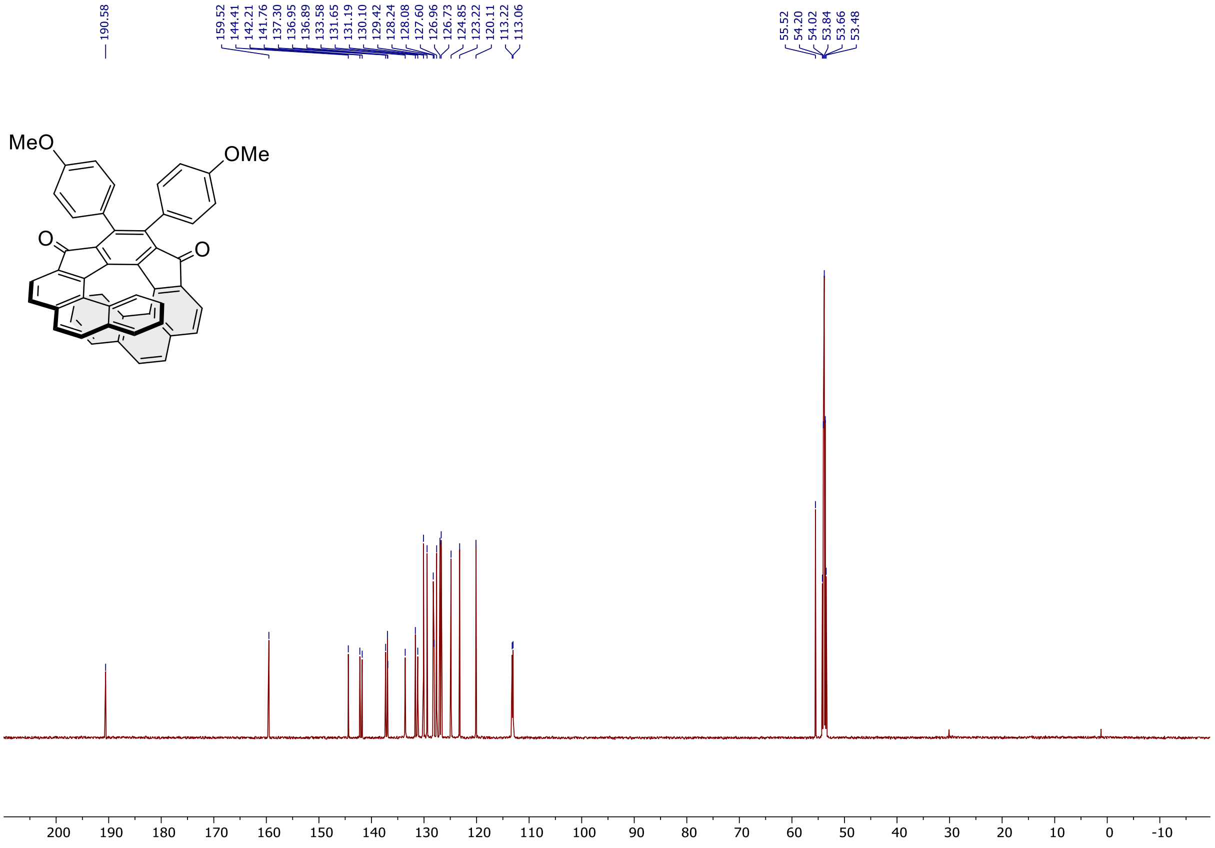

6-(4-methoxyphenyl)-5H-benzo[*no*]indeno[2,1,7,6-*ghij*]naphtho[1,2-*a*]tetraphen-5-one (3).

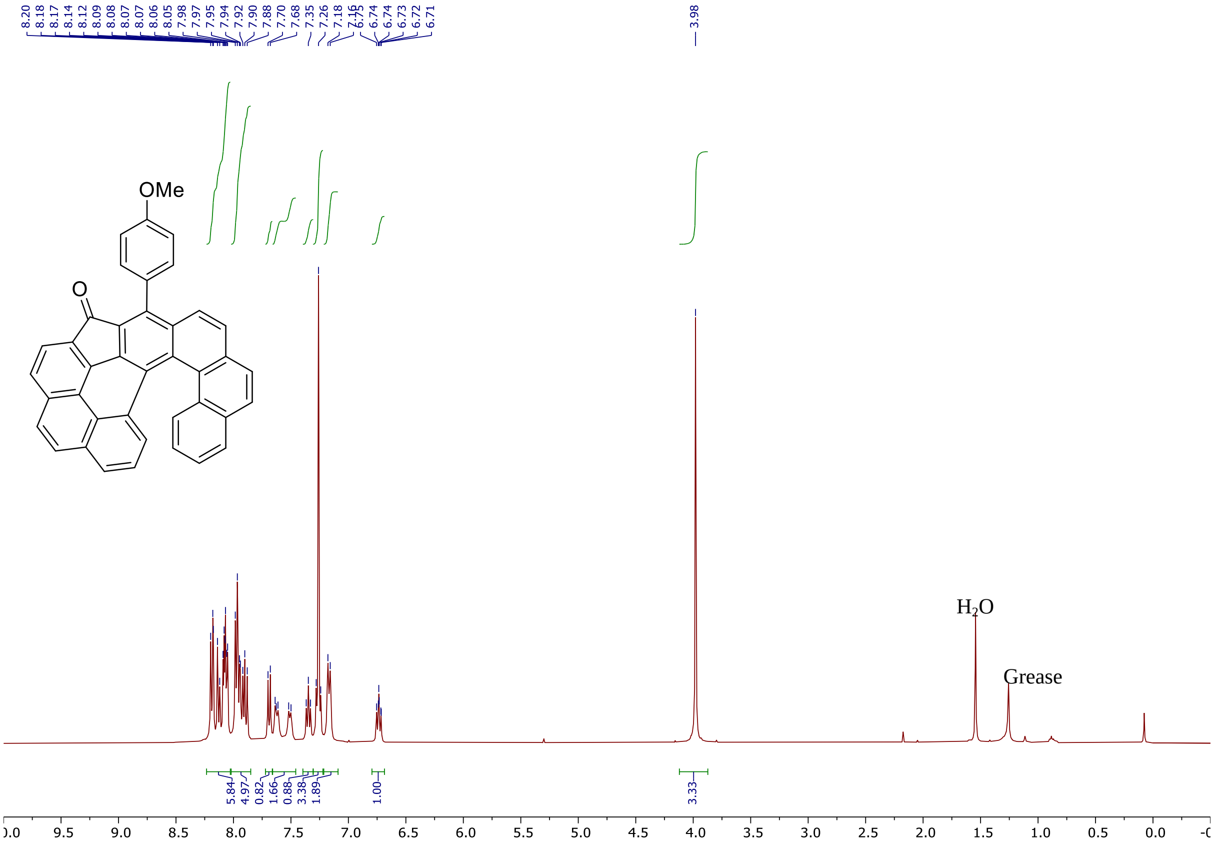

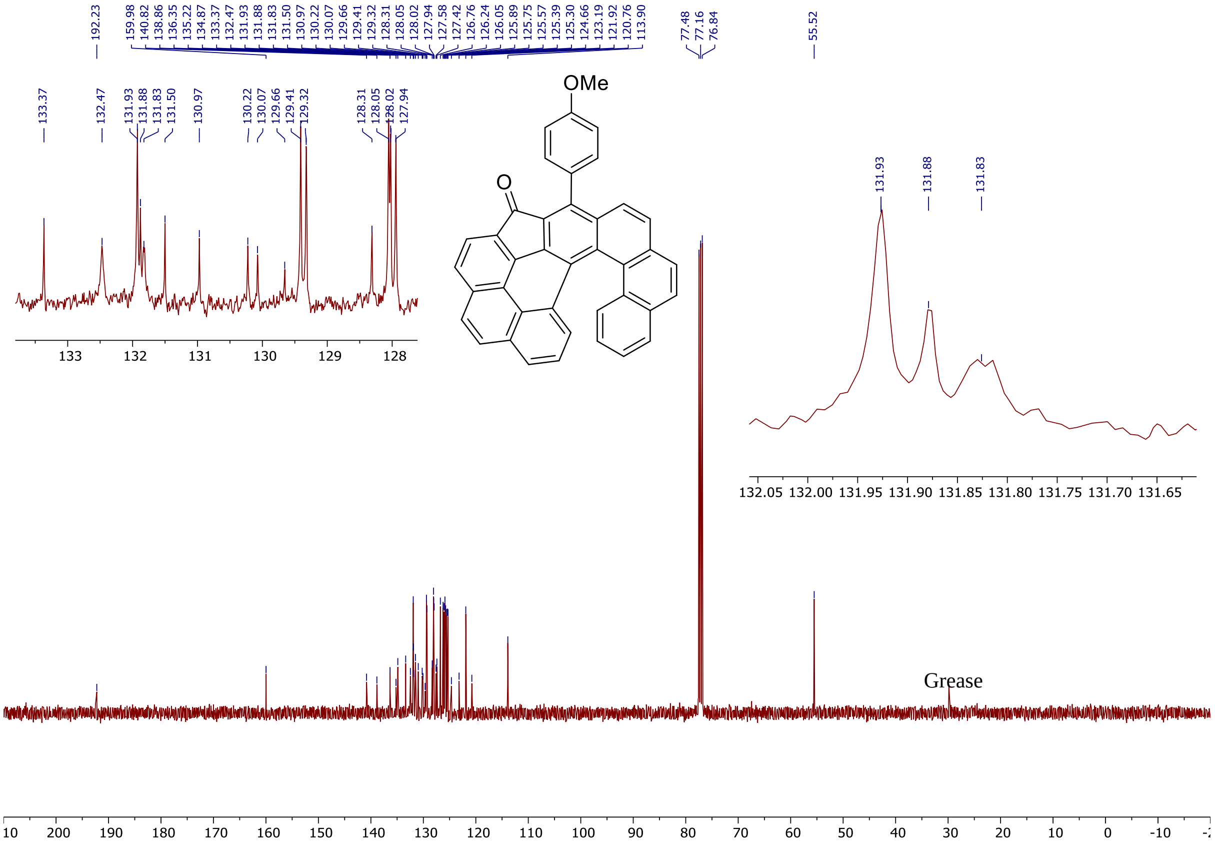

10',11'-bis(4-methoxyphenyl)dispiro[fluorene-9,9'-as-indaceno[2,1-c:7,8-c']diphenanthrene-12',9''-fluorene] (4).

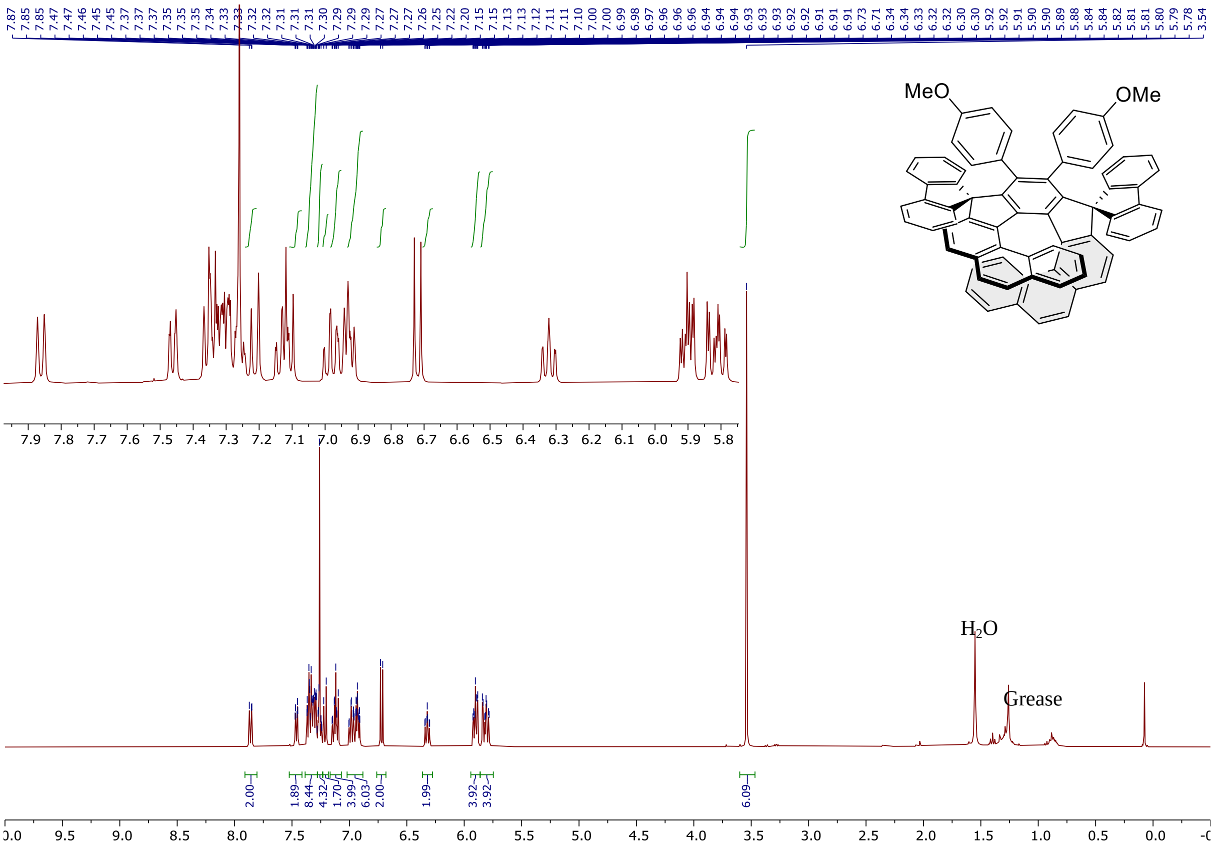

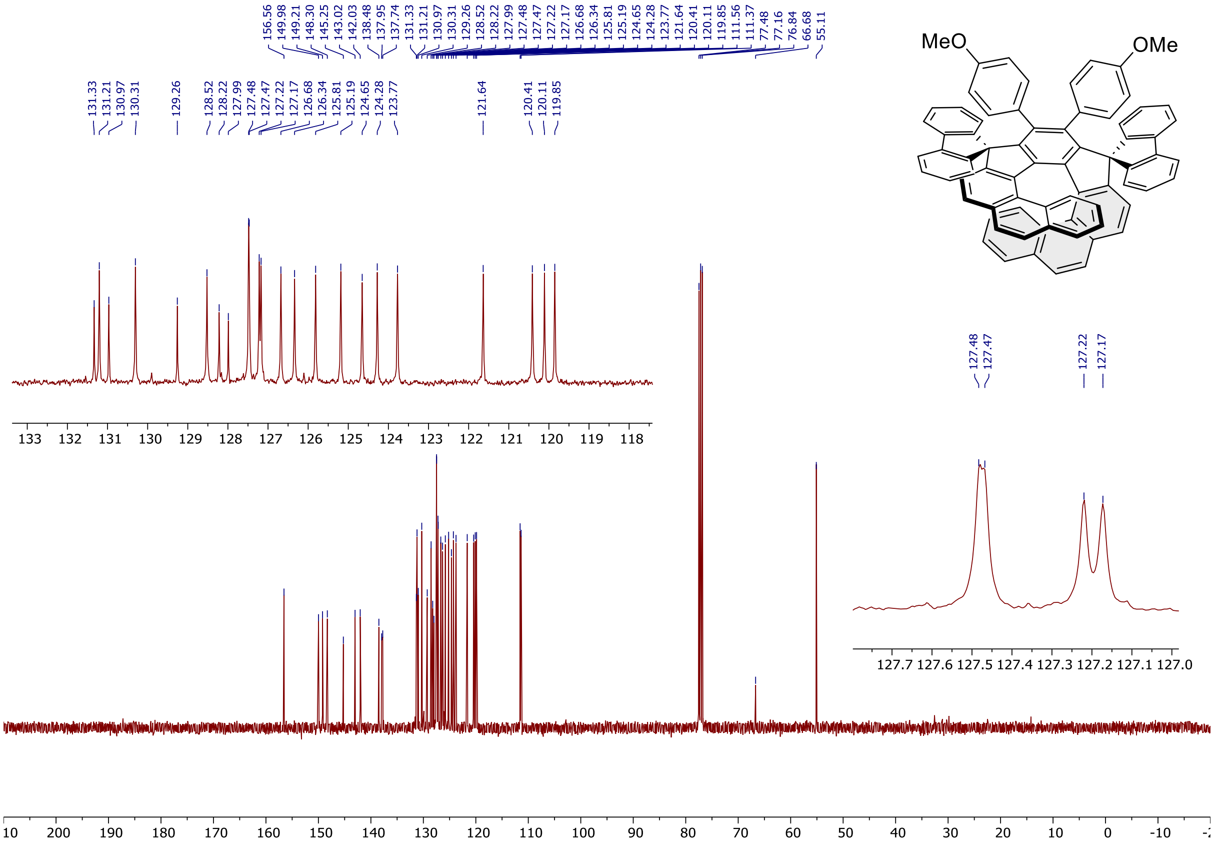

6-(4-methoxyphenyl)spiro[benzo[*no*]indeno[2,1,7,6-*ghij*]naphtho[1,2-*a*]tetraphene-5,9'-fluorene] (5).

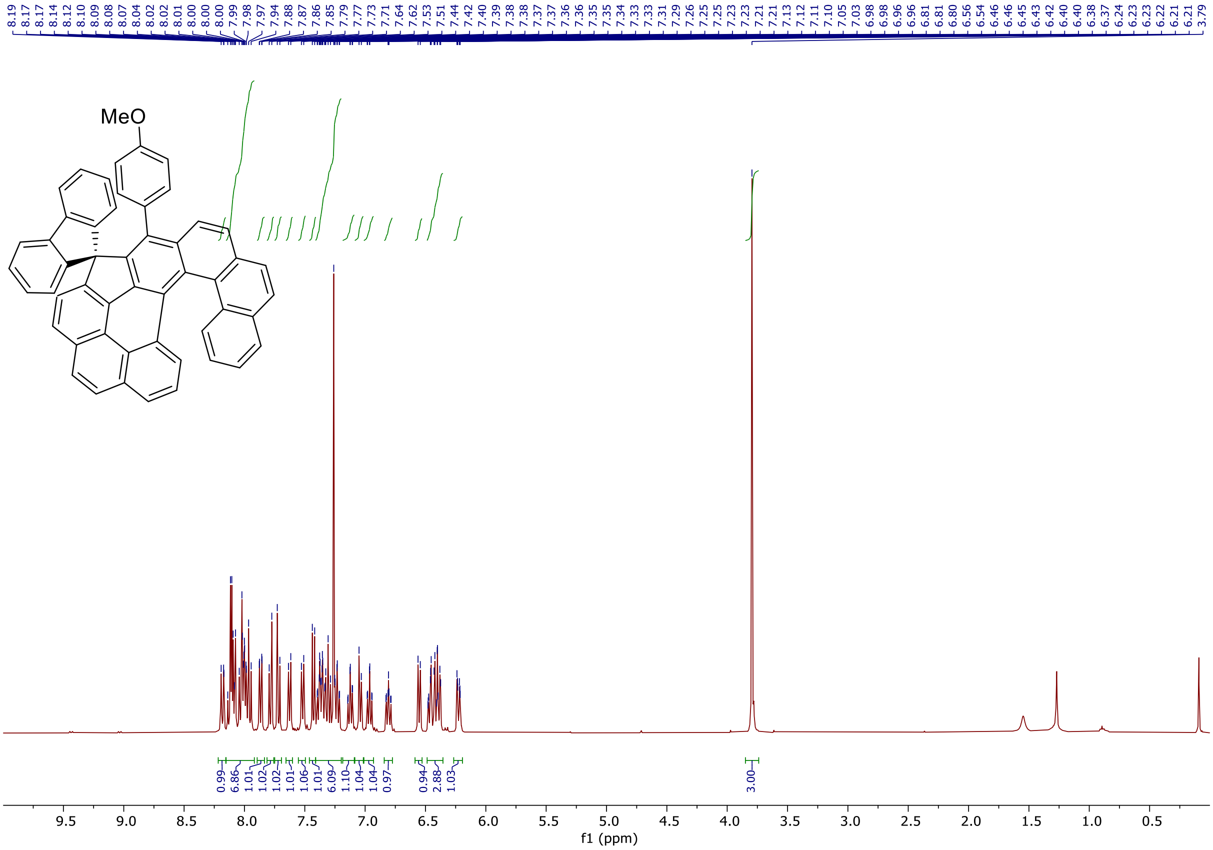

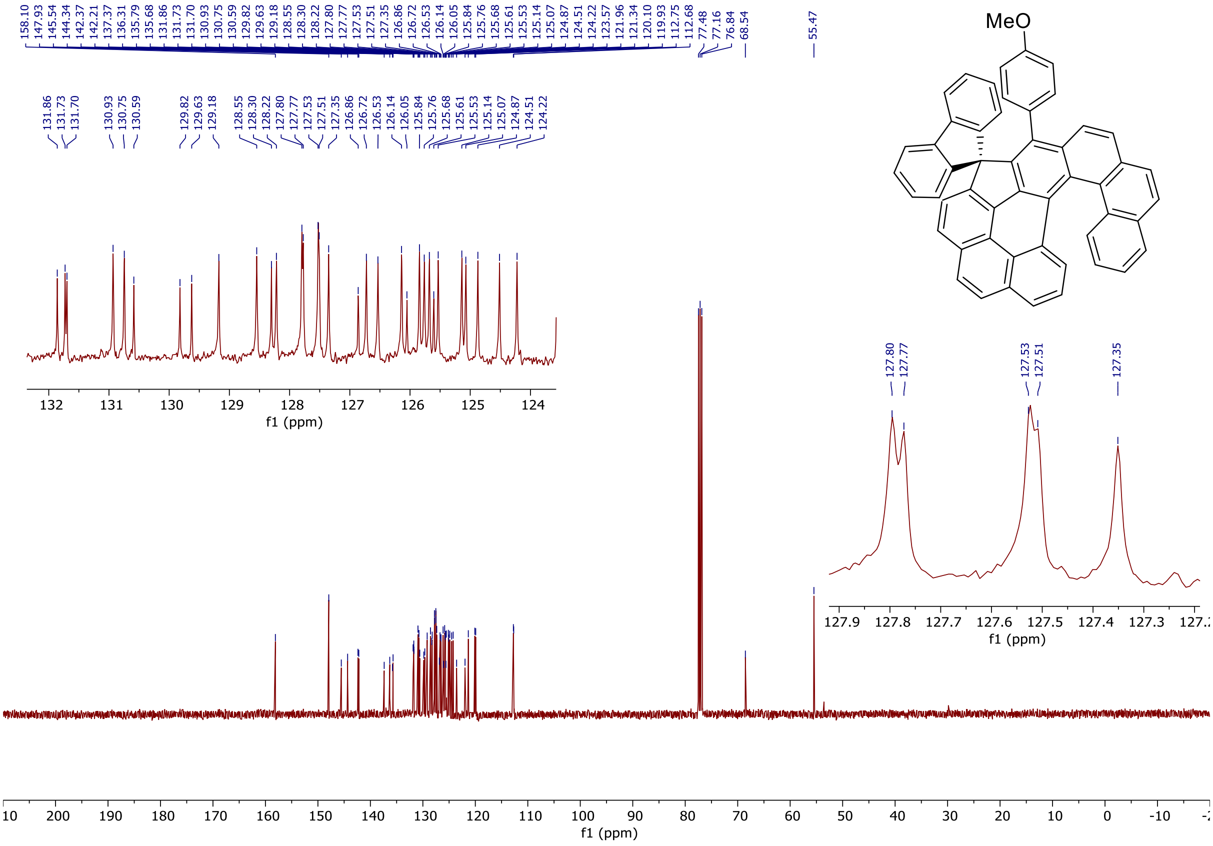

Supplement: Supplementary file 1 — Supporting Information [file CHEM-29-0-s001.pdf]
